# A cautionary tale of low-pass sequencing and imputation with respect to haplotype accuracy

**DOI:** 10.1186/s12711-024-00875-w

**Published:** 2024-01-12

**Authors:** David Wragg, Wengang Zhang, Sarah Peterson, Murthy Yerramilli, Richard Mellanby, Jeffrey J. Schoenebeck, Dylan N. Clements

**Affiliations:** 1 The Roslin Institute and Royal (Dick) School of Veterinary Studies, University of Edinburgh, Easter Bush Campus, Midlothian, EH25 9RG UK; 2 IDEXX Laboratories Inc, One IDEXX Drive, Westbrook, ME 04092 USA

## Abstract

**Background:**

Low-pass whole-genome sequencing and imputation offer significant cost savings, enabling substantial increases in sample size and statistical power. This approach is particularly promising in livestock breeding, providing an affordable means of screening individuals for deleterious alleles or calculating genomic breeding values. Consequently, it may also be of value in companion animal genomics to support pedigree breeding. We sought to evaluate in dogs the impact of low coverage sequencing and reference-guided imputation on genotype concordance and association analyses.

**Results:**

DNA isolated from saliva of 30 Labrador retrievers was sequenced at low (0.9X and 3.8X) and high (43.5X) coverage, and down-sampled from 43.5X to 9.6X and 17.4X. Genotype imputation was performed using a diverse reference panel (1021 dogs), and two subsets of the former panel (256 dogs each) where one had an excess of Labrador retrievers relative to other breeds. We observed little difference in imputed genotype concordance between reference panels. Association analyses for a locus acting as a disease proxy were performed using single-marker (GEMMA) and haplotype-based (XP-EHH) tests. GEMMA results were highly correlated (*r* ≥ 0.97) between 43.5X and ≥ 3.8X depths of coverage, while for 0.9X the correlation was lower (*r* ≤ 0.8). XP-EHH results were less well correlated, with *r* ranging from 0.58 (0.9X) to 0.88 (17.4X). Across a random sample of 10,000 genomic regions averaging 17 kb in size, we observed a median of three haplotypes per dog across the sequencing depths, with 5% of the regions returning more than eight haplotypes. Inspection of one such region revealed genotype and phasing inconsistencies across sequencing depths.

**Conclusions:**

We demonstrate that saliva-derived canine DNA is suitable for whole-genome sequencing, highlighting the feasibility of client-based sampling. Low-pass sequencing and imputation require caution as incorrect allele assignments result when the subject possesses alleles that are absent in the reference panel. Larger panels have the capacity for greater allelic diversity, which should reduce the potential for imputation error. Although low-pass sequencing can accurately impute allele dosage, we highlight issues with phasing accuracy that impact haplotype-based analyses. Consequently, if accurately phased genotypes are required for analyses, we advocate sequencing at high depth (> 20X).

**Supplementary Information:**

The online version contains supplementary material available at 10.1186/s12711-024-00875-w.

## Background

The depth of coverage to which a genome is sequenced accounts not only for the depth but also for the breadth of the genome captured [1]. Given the non-uniformity of whole-genome sequencing (WGS), low-pass sequencing at depths as low as 0.5X captures only a fraction of the genome. The principle behind sequencing at such a low depth is that by leveraging a panel of reference haplotypes, data can be phased and missing genotypes imputed, delivering cost-savings [2]. This strategy depends on several assumptions. First, that the phasing of the reference haplotypes is accurate. Presently, most genotype data are derived from array or short-read sequencing and, in the absence of trios or extended pedigrees, involve statistical phasing that can result in high switch error rates (> 5%), which relates to consecutive heterozygous genotypes being incorrectly phased with respect to one-another [3]. The stochastic nature of many phasing algorithms due to being based on hidden Markov models introduces further variability into phasing accuracy [3]. These switch errors subsequently impact imputation accuracy [4]. Second, imputation assumes that the initial genotypes or genotype likelihoods in the data to be imputed are accurate. A genotype’s likelihood from short-read sequencing is constrained by sequencing depth, given that it is the product of a genotype’s probability over all reads that span the base considered [5]. Finally, the composition of the reference panel of haplotypes can have a significant role on imputation accuracy. For instance, in humans, a population-specific reference panel enriched for African haplotypes outperforms other panels when imputing African American individuals [6]. A similar observation has been reported in cattle, where within-breed and multi-breed reference panels of varying sizes (30 to 150 animals) were evaluated and within-breed panels were found to outperform equally-sized multi-breed panels [7]. That study also reported that a larger multi-breed panel that lacks the subject breed to be imputed, but which included distantly related breeds, returned the same degree of accuracy as a smaller within-breed panel. In dogs, several studies have demonstrated that imputation accuracy is improved when a multi-breed diverse reference panel is used [8, 9]. The size and composition of the reference panel influence the minor allele frequency (MAF) of variants, and it is well established that variants with a low MAF are more difficult to impute accurately, as it is more challenging for imputation algorithms to establish their haplotype background [4, 7, 10].

Domesticated species have different life histories since they have been selectively bred for different purposes. It is important, therefore, to ensure that the reference panel used for imputation is designed to consider a subject breed’s origins. Due to a history of inbreeding, haplotypes within pedigree dogs extend over long distances (up to 100 kb), and portions of these haplotypes are often shared between breeds at varying frequencies [11, 12]. Such a haplotype structure implies that accurate phasing and imputation of genotypes are likely feasible with low-coverage sequencing data. Given that reference-based imputation employs phased haplotypes, we expect accurately phased genotypes to result from the imputation workflow, although this does not appear to have been explored in the literature. With this in mind, we sequenced 30 Labrador retrievers at varying depths of coverage and, using a reference panel of wild canids and dogs from a diversity of breeds, evaluated the impact of sequencing depth on imputation accuracy and downstream association analyses for the chocolate coat colour phenotype. The aim of this study was to inform on the potential utility of low-pass sequencing and imputation in companion animals, and to evaluate the resulting haplotype accuracy. However, our results will likely apply to any system where sample sizes and family information are limited, and where haplotype inference is important.

## Methods

### Sample collection and whole-genome sequencing

We sampled 30 Labrador retrievers using saliva collection kits and isolated DNA according to manufacturer protocols (PERFORMAgene PG-100, DNA Genotek Inc.). Library preparation (TruSeq DNA PCR-free, 150 bp paired-end, 350 bp insert size) and WGS (NovaSeq 6000) of DNA were performed by Novogene (UK). Libraries were sequenced at approximately 50X depth of coverage (calculated as 43.5X post-alignment processing), and also resequenced at approximately 4X (calculated as 3.8X post-alignment processing) and 1X (calculated as 0.9X post-alignment processing) depths of coverage [see Additional file 1: Table S1]. Raw FASTQ files were preprocessed with the fastp v0.21.0 software [13] using default settings to remove short and low-quality reads (length < 15, or base phred quality < 15 over 40% of bases), read pairs where one read has > 5 N bases, and to trim polyG tails (minimum tail length = 10) and adapter sequences. The filtered FASTQ files were aligned to the complete Labrador retriever genome assembly ROS_Cfam_1.0 (NCBI GenBank assembly accession: GCA_014441545.1) using the bwa-mem2 algorithm [14] and duplicates were marked with the GATK v4.2.0.0 [15] MarkDuplicates tool. These sequence processing steps are implemented in BAGpipe (https://bitbucket.org/enzo_tale/bagpipe). Alignment metrics were calculated with bamUtil stats [16].

### Imputation reference panels

Publicly available sequence data for a diverse panel of dogs collated and processed for another ongoing project were used as a reference panel for imputation. These data were aligned to ROS_Cfam_1.0, and variants were called using the Strelka2 software [17]. The resulting genome variant call files (g.VCFs) were merged using Illumina’s gvcfgenotyper to generate a single VCF file for each autosome. A series of filtering steps were applied to retain only high-quality samples and variants [see Additional file 2: Figure S1]. From an initial 1706 samples and more than 1.4 billion variants, a final dataset of 1021 samples and 9.2 M (0.66%) variant records were retained [see Additional file 1: Table S2]. The variants included multiallelic single nucleotide variants (SNVs) and insertion-deletion mutations (INDELs) that were decomposed into biallelic records using the bcftools program [18]. Two subset panels were derived from this ‘full’ reference panel. Panel 1 comprised 21 Labradors and 235 randomly selected samples (no more than 1 per breed or wild species). Panel 2 replaced 20 of the Labradors from panel 1 with randomly selected samples, ensuring no more than two per breed and wild species. The wild canids within the dataset included representatives of coyote, dingo, and wolf. Sporadic missing variants were imputed with BEAGLE v5.4 [19, 20].

### Down-sampling of sequence data and genotype imputation

Alignment files (BAM) for the Labrador retrievers that were sequenced at 43.5X in this study were down-sampled using the sambamba v0.7.1 software [21] to 9.6X and 17.4X depths of coverage by retaining 20 and 40% of reads, respectively. Sequencing depths were subsequently determined with samtools coverage v1.10 [22]. For each dataset (43.5X, 17.4X, 9.6X, 3.8X, 0.9X), genotype likelihoods (GL) were calculated using bcftools, as outlined in the GLIMPSE pipeline [23] documentation (https://odelaneau.github.io/GLIMPSE). Target sites for imputation were the 9.2 M variants in the full reference panel. GLIMPSE_chunk was used to define 20-Mb windows for imputation. GLIMPSE_phase was used to genotype GL and to phase genotypes using their respective reference panel. GLIMPSE_ligate was used to combine the chunks along each chromosome. In all cases, GLIMPSE v1.1.1 static binaries were used. Following imputation, genotypes with a genotype probability (GP) lower than 0.95 were set to missing and phased variants were extracted using GLIMPSE_sample. Additional file 3: Figure S2 illustrates the workflow from DNA extraction through to imputation.

### Concordance analyses

Concordance between genotypes sequenced at different depths of coverage relative to those at 43.5X depth, prior to imputation, were performed using the bcftools gtcheck tool. Post-imputation, concordance was evaluated using GLIMPSE_concordance by comparing post-imputation genotypes from each reference panel to the 43.5X depth pre-imputation genotypes. Sites were evaluated if they had a minimum posterior probability of 0.9999 and a minimum depth of 1, 2, 5, and 10, for sequencing depths of 0.9X, 3.8X, 9.6X, and 17.4X, respectively. Additional file 4: Figure S3A illustrates the inputs for the two concordance evaluations. Discordance between post-imputation genotypes from each sequencing depth relative to pre-imputation genotypes at 43.5X depth was further analysed using bcftools gtcheck. Haplotype discordance was evaluated by splitting phased diploid genotypes into separate VCF files per haplotype and comparing equivalent haplotypes between each sequencing depth and those at 43.5X. All statistical analyses of concordance results were performed in R.

### Association analyses

We used chocolate coat colour as a proxy for a disease phenotype in the Labrador retrievers. Linear mixed model association analyses for the phenotype were performed with GEMMA v0.98.5 [24], accounting for genomic kinships and sex. Of the dogs that we sequenced, six were chocolate coloured, two were fox red, two were yellow, and 20 were black [see Additional file 1: Table S2]. The input files for GEMMA were prepared using Plink v1.90p [25] and a MAF filter of 0.01 was applied by GEMMA during the analyses [see Additional file 4: Figure S3B]. On average, 4.5 M ± 173 K variants were analysed for each sequencing depth and reference panel dataset. We also performed a haplotype-based analysis based on cross-population extended haplotype homozygosity (XP-EHH), which was performed using the hapbin software [26]. The input files for hapbin were prepared with Plink and bcftools. We used the default EHH (0.05) and MAF (0.05) cutoffs implemented in hapbin. All statistical analyses on the GEMMA and hapbin results were performed in R.

## Results

### Whole-genome sequencing of canine saliva DNA requires over-estimation of targeted depth

DNA isolated from saliva swabs of 30 Labrador retrievers was whole-genome sequenced on the NovaSeq platform, generating a mean data volume and base quality Q30 of 169.74 Gb and 92%, respectively [see Additional file 1: Table S2]. Sequencing reads were aligned to the Labrador retriever assembly (ROS_Cfam_1.0), returning a mean mapping ratio of 0.8, which was not significantly correlated with raw read count (F-statistic p = 0.4). The mean observed depth of coverage (43X) was on average 30% lower than the expected depth of coverage (56X) based on the volume of data generated and the size of the assembly (2.4 Gb). To investigate the effects of low-coverage sequencing on genotype accuracy, imputation concordance, and downstream analyses, we resequenced the same libraries at lower depths of coverage, aiming to achieve depths of 4X and 1X. We generated on average 13.5 Gb and 3 Gb data per dog at these respective depths, each returning mean Q30 base qualities of 91% and a mapping ratio of 0.8. Given these sequencing depths and mapping ratios, we expected to achieve 4.5X and 1X depths of coverage, but instead observed depths of 3.75X and 0.87X [see Additional file 1: Table S1]. Considering that mapping ratios remained consistent across the different sequencing depths, the observed/expected depth ratios suggest that sequencing at higher depths captures relatively more off-target (potentially bacterial) sequences compared to sequencing at lower depths. Differences in average observed/expected depth ratios between the three depths of coverage were significant (Kurksal-Wallis p = 5.7 × 10^–16^). These results indicate that whole-genome sequencing DNA isolated from saliva requires a higher sequencing depth than targeted. The regression slope of expected versus observed depths of coverage was 1.3, which we suggest using as a guide for scaling (e.g. to achieve 30X depth coverage post-mapping, a library should be sequenced to generate ~ 39X raw data coverage).

**Fig. 1:**
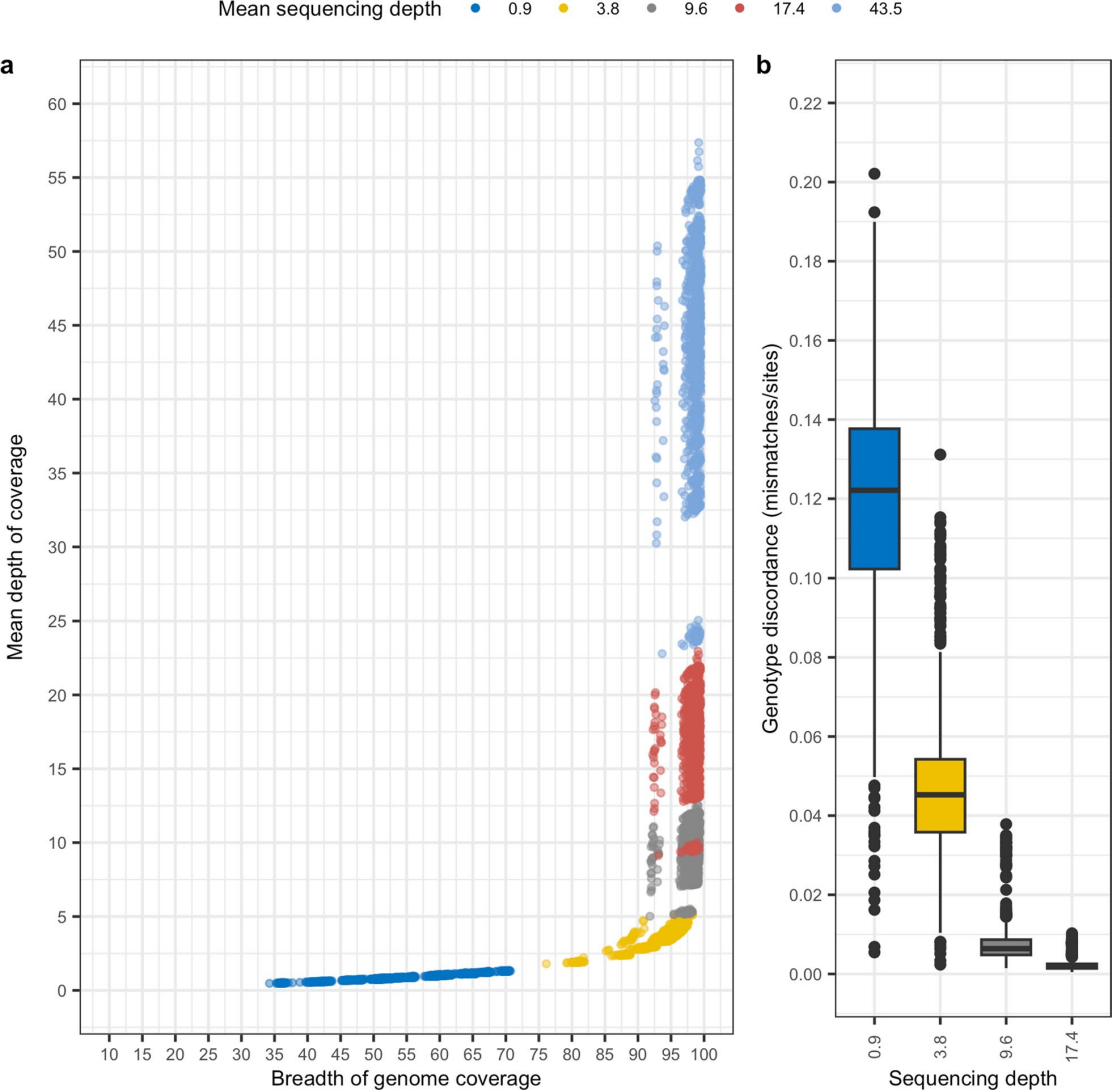
Sequencing depth versus breadth of genome coverage and genotype discordance. **a** Depth of coverage is plotted against breadth of coverage for Labrador retriever autosomes from each sequencing depth. The shift in breadth of coverage observed at all depths is associated with the inability to map to unresolved regions of the assembly on chromosome 26 (e.g. 26:25818821–26738102). **b** Pre-imputation genotype discordance for genotypes calculated at each sequencing depth relative to genotypes calculated at 43.5X

We next sought to evaluate the impact of sequencing depth on genotype concordance. The alignment data from libraries sequenced at 43.5X depth were down-sampled in silico to approximately 10X and 20X depths of coverage. For the 38 dog autosomes, sequencing depths were recalculated using samtools coverage and genotypes were called using bcftools GL for the 9.2 M variant records in the full reference panel [see Additional file 3: Figure S2]. The mean sequence depths reported across the various datasets were 0.9X, 3.8X, 9.6X, 17.4X and 43.5X, which will be used to refer to the different datasets in the following. As expected, breadth of coverage increased with depth of coverage, with diminishing returns at depths ≥ 3.8X (Fig. 1a). Sequencing depths ≥ 9.6X had a mean breadth of coverage of 98.5 ± 1.3%, while at 3.8X the mean breadth was 94.2 ± 3.39%, and at 0.9X it was 54.1 ± 7.9%. The correlation between sequencing depth and the ratio of discordant genotypes relative to those calculated at 43.5X depth was significant (Fig. 1b; Pearson’s product-moment correlation p < 0.05, *r* = − 0.8). At sequencing depths ≥ 9.6X, mean concordance was higher than 99%, while at 3.6X and 1X mean concordances were 95 and 88%, respectively. These results indicate that sequencing as low as 3.6X depth of coverage captures approximately 95% of the genome with high accuracy.

### Reference panel size has a negligible impact on imputation accuracy in Labrador retrievers

**Fig. 2:**
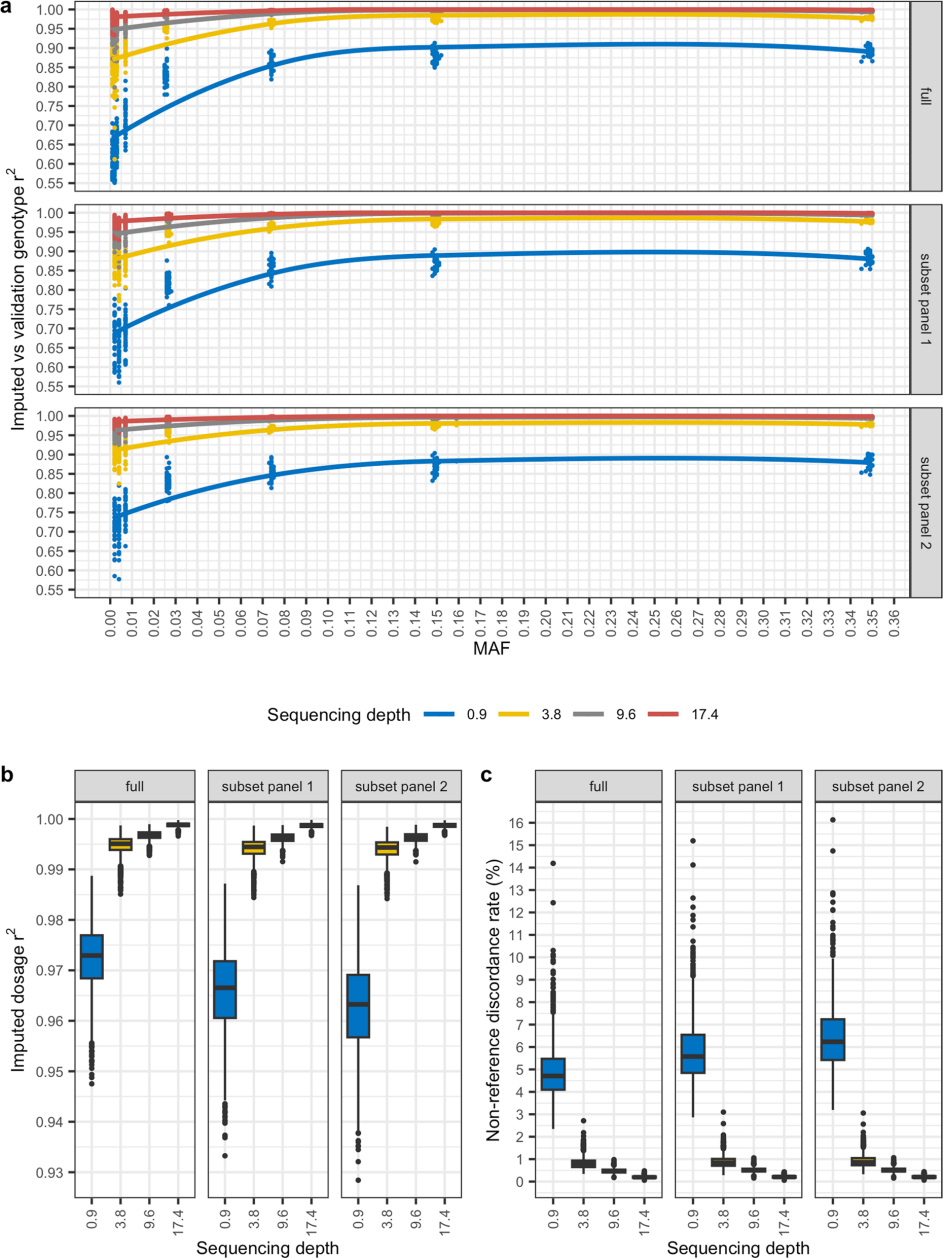
Post-imputation genotype *r*^2^, dosage *r*^2^, and non-reference allele discordance rate (%) following imputation at each depth of coverage, based on different reference panels, relative to high coverage. **a** MAF-binned *r*^2^ values comparing imputed versus validation genotypes. **b** Box plots of imputed dosage *r*^2^ values for imputed genotypes from each sequencing depth. **c** Box plots of non-reference discordance rate (%) for imputed genotypes from each sequencing depth. In each case, the validation genotypes are the 43.5X imputed genotypes

For each sequencing depth, phasing and imputation were performed with GLIMPSE using three reference panels. The ‘full’ panel comprised 1021 dogs [see Additional file 1: Table S3], from which two subsets of 256 dogs each were generated. The two subsets differed by 21 dogs, with panel 1 including 21 Labrador retrievers, while panel 2 substituted 20 of the Labrador retrievers for randomly sampled dogs. A summary of variant counts binned by allele frequency for each reference panel and for the genotypes imputed from each depth of coverage based on the full panel is provided in Additional file 1: Table S4. Concordance was calculated between the post-imputation genotypes that resulted from each reference panel and the 43.5X pre-imputation genotypes. Imputed genotype *r*^2^ values reported by the GLIMPSE concordance tool were remarkably consistent for the different depths of coverage across the three reference panels (Fig. 2a; median genotype *r*^2^ at 17.4X = 0.99 ± 0.22 sd, 9.6X = 0.98 ± 0.23, 3.8X = 0.94 ± 0.23, 0.9X = 0.79 ± 0.23). Regressing imputed genotype *r*^2^ on MAF, with depth and reference panel as covariates, reported each of the regression coefficients to be significantly different from 0 [see Additional file 1: Table S5]. SNV imputed dosage *r*^2^ was also significantly correlated with sequencing depth (Fig. 2b; Pearson’s product-moment correlation p < 0.05, r = − 0.68), and regressing imputed dosage *r*^2^ on depth with reference panel as a covariate also resulted in each regression coefficient to be significantly different from 0 [see Additional file 1: Table S6].

Non-reference allele discordance rates (Fig. 2c), also reported by the GLIMPSE concordance tool, largely mirrored the imputed dosage *r*^2^ results, with discordance being negatively correlated with sequencing depth (Pearson’s product-moment correlation p < 0.05, *r* = − 0.68). An analysis of median genotype mismatch rates for SNVs indicated that heterozygous genotypes accounted for most mismatches across all depths of coverage and reference panels used [see Additional file 1: Table S7]. It is worth noting that the median homozygous reference allele mismatch rate was less than 1% for all depths of coverage, and the median homozygous alternate allele mismatch rate was less than 1% for all depths except for 0.9X, for which it was 2.9 ± 1.16 when using the full reference panel, 3.5 ± 1.5 when using panel 1, and 3.9 ± 1.5 when using panel 2. This is not unexpected, as sequencing at low depths reduces the likelihood of generating a sufficient number of reads that span both alleles to confidently assign a heterozygous genotype.

### Single-marker association analysis results are highly consistent across sequencing depths

**Fig. 3:**
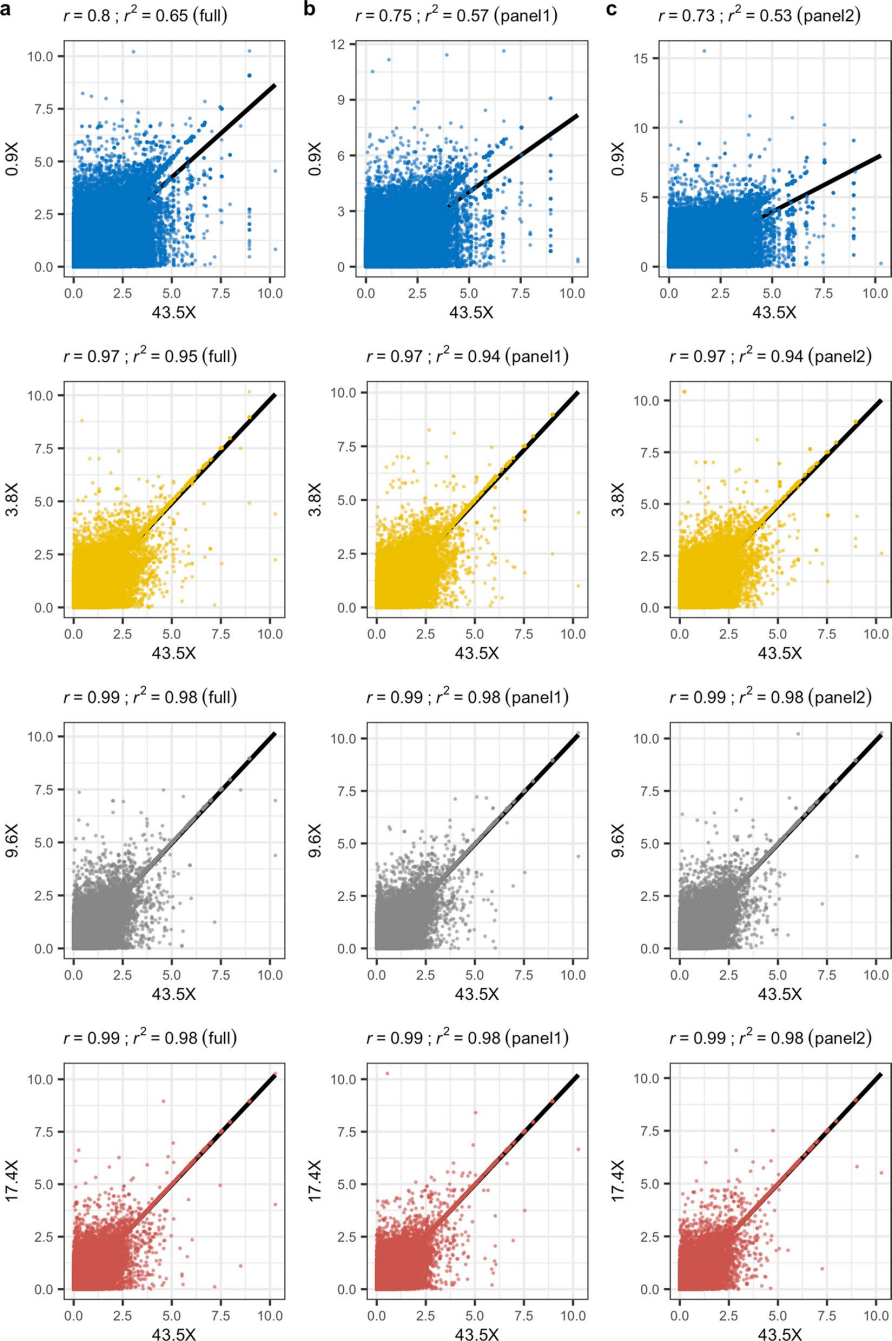
Biplots of -log10(p) values from GEMMA analyses at each sequencing depth relative to 43.5X. **a** Results based on using the full reference panel for imputation. **b** Results based on using panel 1 for imputation. **c** Results based on using panel 2 for imputation. In each case, the black line indicates the linear fit of the two datasets, with the *r* and *r*^2^ value included at the top of the plot

To evaluate the impact of genotype imputation on association analyses, we applied a linear mixed model using GEMMA to identify a genetic association with chocolate (brown) coat colour phenotype, as a proxy for disease, which was present in six of the 30 dogs that we sequenced [see Additional file 1: Table S1]. The chocolate coat colour in Labrador retrievers is due to recessive alleles at the *tyrosinase related protein 1* (*TYRP1*) gene, while carriers of the dominant allele have a black coat [27]. Several mutations in this gene have been associated with brown coat colour in dogs, and at least three independent mutations have been identified in Labrador retrievers [27]. The analysis was performed on the phased genotypes that resulted from the GLIMPSE pipeline for each depth of coverage and reference panel, applied a MAF filter of 0.01, and accounted for sex as a covariate. Of the 9.2 M variants provided, GEMMA analysed 4.5 M ± 173 K variants on average across the various datasets. Relative to the results obtained at 43.5X, we observed a strong correlation (Pearson’s *r* ≥ 0.97) of p values for depths ≥ 3.8X, while at 0.9X a notable reduction was found (*r* ≤ 0.8) (Fig. 3). For reference, Manhattan plots of the GEMMA results based on the full reference panel and panels 1 and 2 are provided in Additional file 5: Figure S4, Additional file 6: Figure S5 and Additional file 7: Figure S6, respectively.

**Fig. 4:**
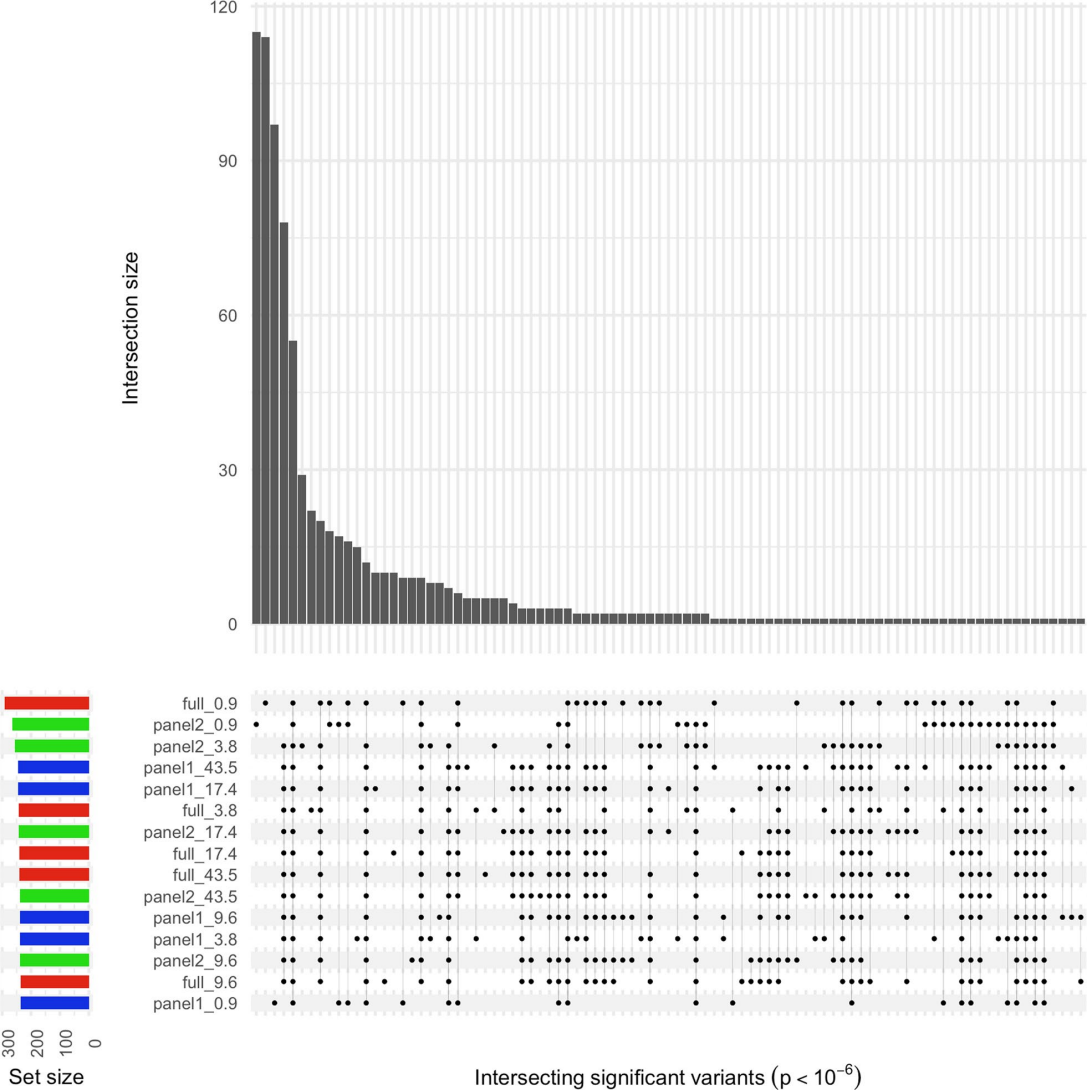
UpSet plot showing intersection of significant GEMMA p values between the different sequencing depths. Results are presented from each depth of coverage, imputed using each reference panel. The main bar plot indicates the count of intersecting significant variants (p < 1 × 10^–6^) between the datasets highlighted with points in the matrix beneath. Each row in the matrix corresponds to a specific dataset, and the label indicates the reference panel used (full, panel 1, or panel 2), and the sequencing depth of the data (0.9X, 3.8X, 9.6X, 17.4X, or 43.5X). The bar plot to the left indicates the number of significant variants identified for the corresponding dataset and is coloured according to the reference panel used

The intersection of significant p values (p < 1 × 10^–06^) from the GEMMA analysis across the different depths of coverage and reference panels used for imputation was large (Fig. 4), with 88% of all possible combinations of dataset pairs being significantly correlated (Pearson’s product-moment correlation p < 0.05). Subset panel 2 at depth 0.9X featured in 12 of the 13 pairs that were not significantly correlated.

### Erroneous phasing impacts haplotype-based analyses

Genotype imputation accuracy is positively correlated with MAF (Fig. 2a) and has been shown to be influenced by the size of the reference panel, variant density, haplotype accuracy, and sequencing coverage [10]. The imputation and GEMMA results presented here were highly consistent between reference panels of different sizes (1021 and 256), particularly at sequencing depths ≥ 3.8X. However, the concordance and GEMMA analyses are uninformative with respect to haplotype accuracy, as they consider each variant independently. Genotype imputation depends on the identification of shared haplotype segments between the reference panel and the subject and, therefore, any phasing error can result in the fragmentation of shared haplotype segments [10].

To explore the impact of potential phasing errors in more detail, we undertook association analyses for chocolate coat colour using the XP-EHH method for each depth of coverage and reference panel. Standardised (Z-score) XP-EHH values indicate the extent of haplotype fixation within a population and can be positive or negative depending on whether selection is in the direction of population A or B. Here, population A comprised the 24 dogs that did not have a chocolate coat colour, while population B comprised the six dogs that did. For reference, Manhattan plots of the XPEHH results based on the full reference panel and panels 1 and 2 are provided in Additional file 8: Figure S7, Additional file 9: Figure S8, and Additional file 10: Figure S9, respectively.

**Fig. 5:**
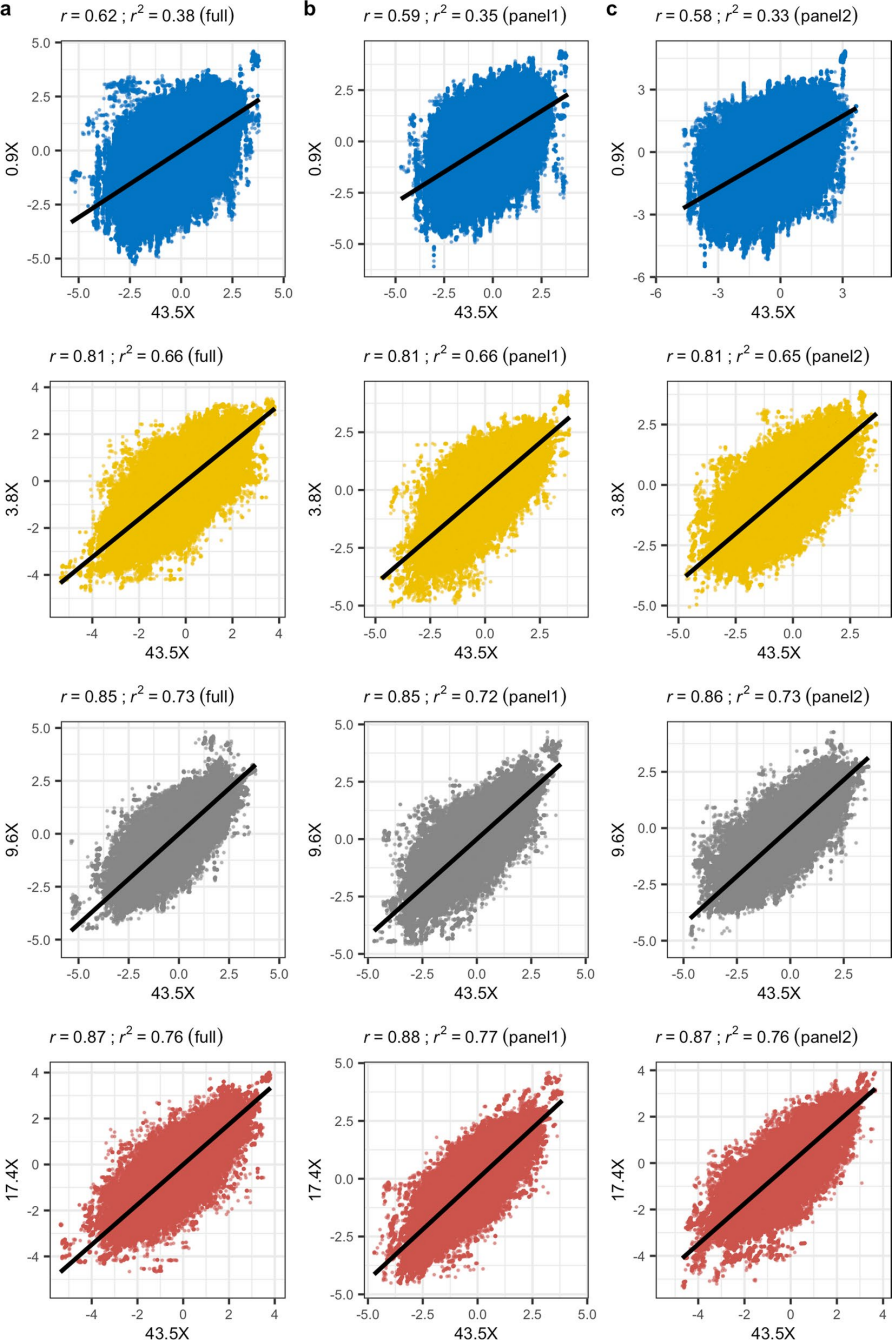
Biplots of Z-scores from XPEHH analyses at each sequencing depth relative to 43.5X. **a** Results based on using the full reference panel for imputation. **b** Results based on using panel 1 for imputation. **c** Results based on using panel 2 for imputation. In each case, the black line indicates the linear fit of the two datasets, with the *r* and *r*^2^ value included at the top of the plot

**Fig. 6:**
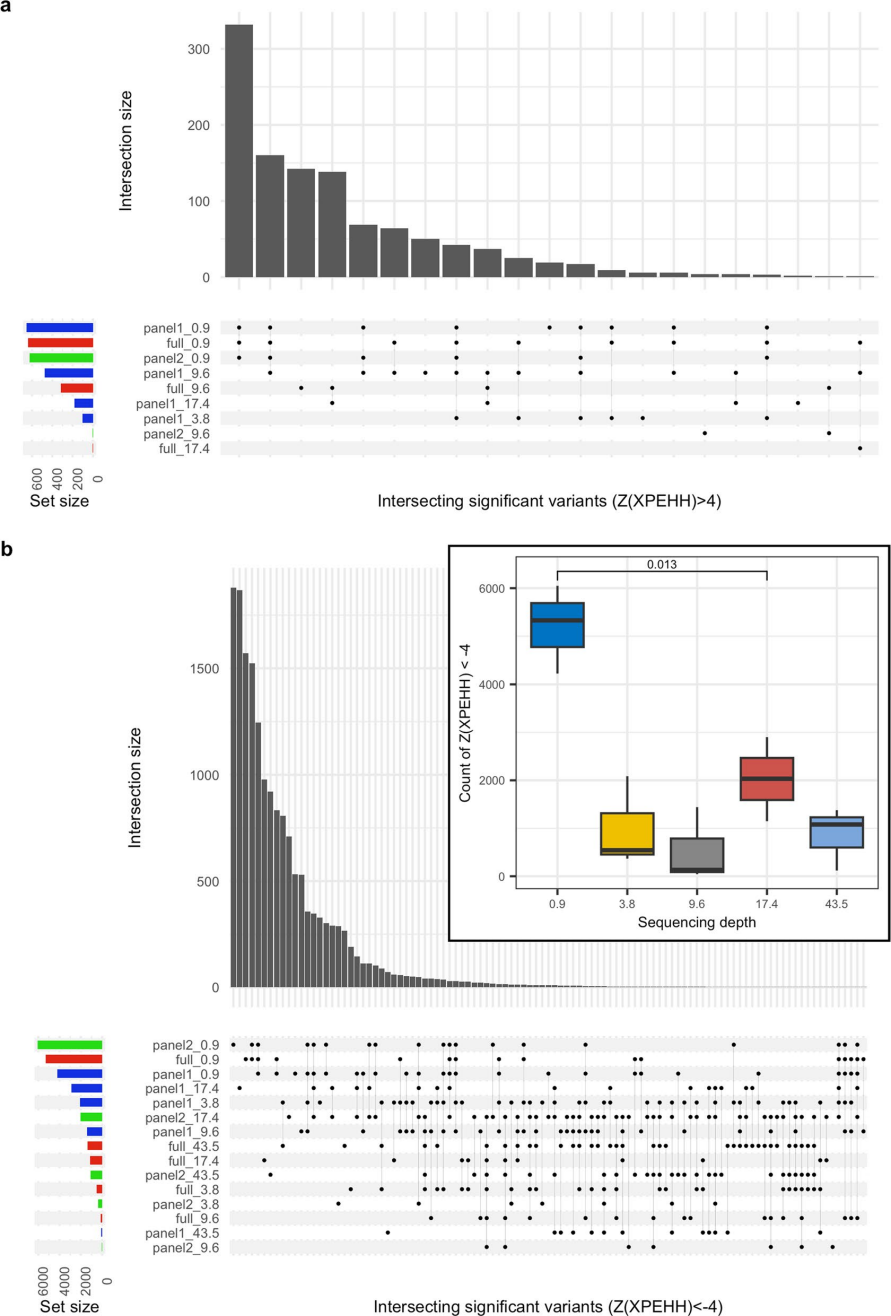
UpSet plot showing the intersection of significant XPEHH Z-scores between the different sequencing depths. **a** Results are presented from each depth of coverage, imputed with each reference panel, for Z-scores > 4. **b** Results are presented from each depth of coverage, imputed with each reference panel, for Z-scores < − 4. For both panels, the main bar plot indicates the count of intersecting significant variants between the datasets highlighted with points in the matrix beneath. Each row in the matrix corresponds to a specific dataset and the label indicates the reference panel used (full, panel 1, or panel 2), and the sequencing depth of the data (0.9X, 3.8X, 9.6X, 17.4X, or 43.5X). The bar plot to the left indicates the number of significant variants identified for the corresponding dataset, and is coloured according to the reference panel used. If no significant variant was identified for a dataset then it was not included in the matrix. The inset in panel **b** shows a box plot of Z-scores < − 4 for each sequencing depth, with a T test p value comparing the means of the two highest counts (0.9X and 17.4X)

Relative to the results for the 43.5X coverage, we observed a good correlation (Pearson’s *r* ≥ 0.81) for Z-scores at depths ≥ 3.8X (Fig. 5), while the correlation was notably weaker at 0.9X (*r* ≤ 0.62). The number of unique variants with significant positive Z-scores (n = 1131) was considerably smaller than the number with significant negative Z-scores (n = 17,260; Fig. 6). We also observed that no significant positive Z-scores resulted from the 43.5X dataset, which is considered the validation dataset in these analyses. This is as expected because we did not expect to observe selection in non-chocolate coat colour dogs when testing for this phenotype. By contrast, all other datasets returned significant negative Z-scores, which is consistent with selection in these dogs for chocolate coat colour. The correlation between the number of significant negative Z-scores and depth of coverage was significant (Pearson’s product-moment correlation p < 0.05, *r* = − 0.59). However, this was driven by the 0.9X results, which if excluded resulted in a non-significant correlation (p = 0.67, *r* = 0.14). This was further illustrated by comparing the counts between 0.9X, which returned the most results (mean across panels = 5199 ± 922), and 17.4X, which returned the second most (2026 ± 877), with a two-sample t-test (p = 0.013; Fig. 6b inset). This implies that sequencing at very low depth (< 1X) returns a large number of false positives. While there was considerable intersection of significant variants across GEMMA analyses for the different depths of coverage and reference panels (Fig. 4), this was not the case for significant Z-scores from the XPEHH analyses (Fig. 6).

**Fig. 7:**
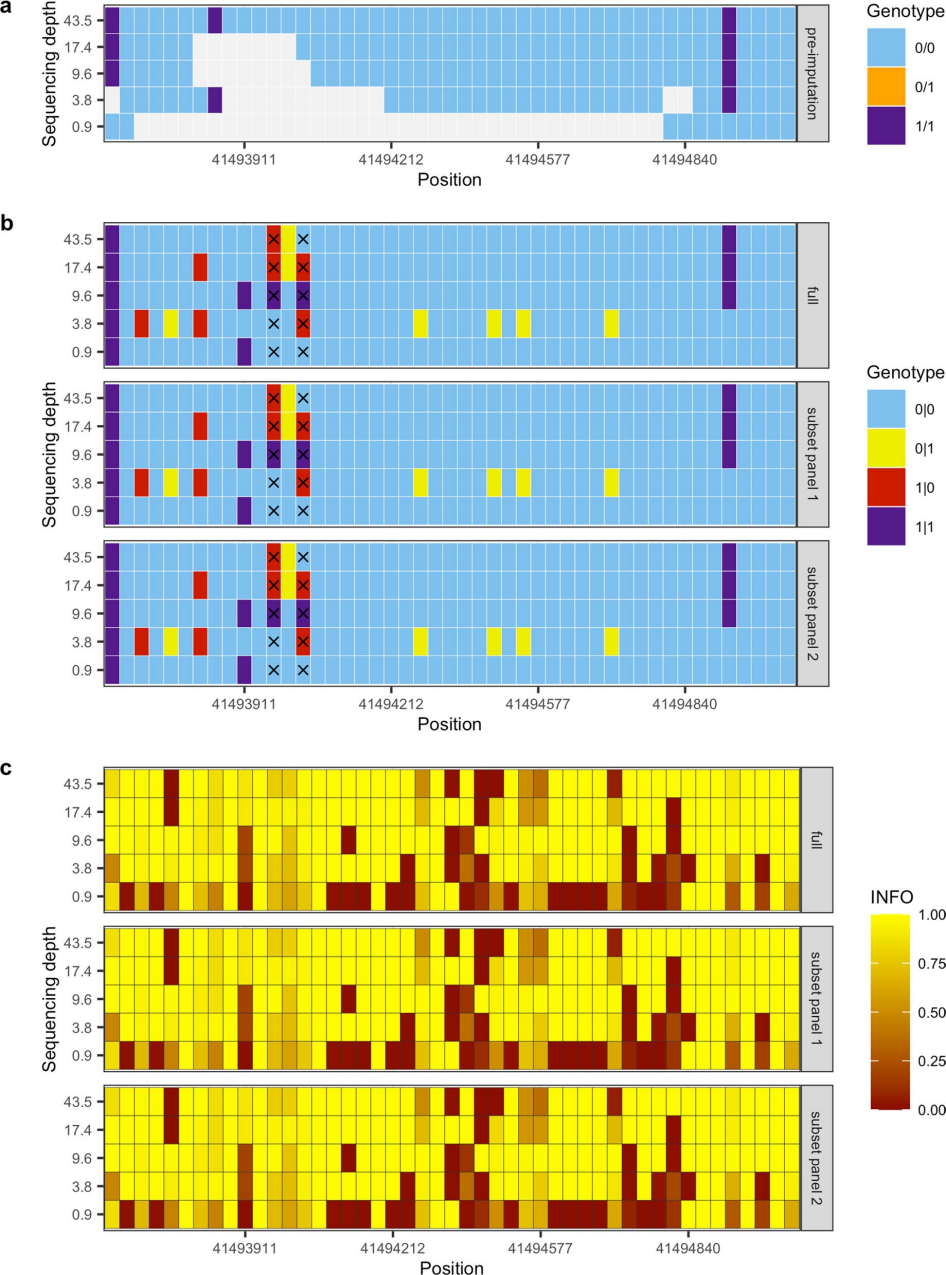
Example of the impact of sequencing depth on genotype imputation accuracy. **a** A matrix of pre-imputation genotypes for a single dog (LAB_11) for the genomic region 18:41493468–41495040. **b** A matrix of post-imputation genotypes for the same region and dog for each of the reference panels used. The genotypes are consistent across reference panels, but differ at some variants across sequencing depths, for instance the two variants annotated with X each return three possible genotypes. **c** A matrix of variant IMPUTE INFO scores for the same region and dog for each of the reference panels used. The INFO score is on a scale of 0 to 1, with 1 indicating a high certainty of imputed genotype accuracy

We next sought to explore the underlying haplotype structure in a region that was suspected to be a false positive signal of selection, the purpose of which was to identify the underlying factors. One such region, 18:41493468–41510,323 exhibited significant positive Z-scores (> 4) only at depths of 9.6X and 17.4X [see Additional file 8: Figure S7 and Additional file 9: Figure S8]. Genotypes in this region were retrieved for each depth of coverage and reference panel. Figure 7a illustrates the genotypes for one dog (LAB_11) prior to applying the imputation workflow, showing that inconsistent genotypes relative to the other sequencing depths were only observed for the 0.9X depth. After applying the imputation workflow, the same genotypes for the same dog and region showed consistency across the different panels used for imputation, but inconsistency across the various depths, including for instance two variants that each returned three possible genotypes (Fig. 7b). Several genotypes that were not missing prior to imputation had their state changed as a result of imputation, including a variant that was homozygous for the alternate allele at 43.5X depth but was homozygous for the reference allele post-imputation. The imputation workflow returns the IMPUTE INFO quality score for each variant, which is an estimate of imputation quality on a scale of 0 to 1, where 1 indicates that a genotype has been imputed with high certainty. The reported INFO scores for this region were consistent across reference panels used and notably lower at 0.9X relative to other sequencing depths (Fig. 7c). It is also worth noting that the two variants that each returned three possible genotypes across the various depths did not return low INFO scores (> 0.7 across all depths).

**Fig. 8:**
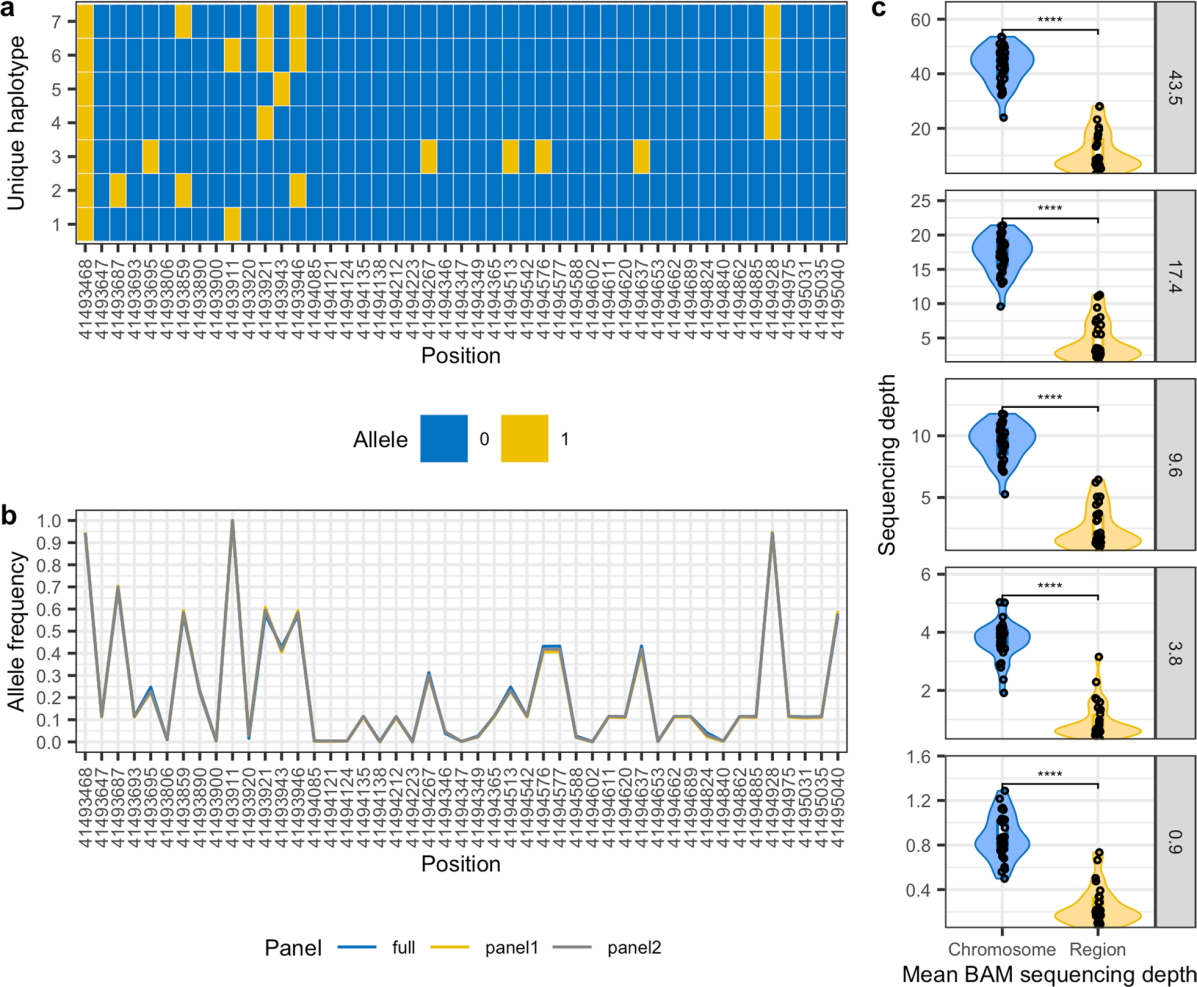
Impact of genotype accuracy on haplotype inference. **a** From the genomic region 18:41493468–41495040 presented in Fig. 7 there are seven possible haplotypes for this dog (LAB_11) across the different depths. **b** The allele frequencies at these variants from the different reference panels used for imputation are highly consistent. **c** The BAM file sequencing depths for all dogs in this region are consistently and significantly lower than the chromosome average for each of the different depths of coverage analysed (paired Wilcox test p < 0.05)

As a consequence of these inconsistencies, seven distinct haplotypes were possible for the 47 variants spanning a 1.5 kb subset of this region (18:41493468–41495040; Fig. 8a). These inconsistencies did not appear to be linked to low reference panel allele frequencies, as the genotype calls that varied across the different sequencing depths coincided with variants with a MAF > 0.1 (Fig. 8b). At all sequencing depths, we observed a significant difference (paired Wilcox test p < 0.05) in the depth of coverage for this region relative to the chromosome as a whole (Fig. 8c). This suggests that genomic regions with reduced coverage, arising for instance because of challenges in read mapping possibly linked to repetitive elements or sequence divergence, may be more challenging to accurately phase.

To determine whether there was a link between the number of haplotypes identified for an individual for a given region across the different sequencing depths with the median XPEHH Z-score for that region, we randomly sampled 10,000 regions throughout the genome [see Additional file 11: Figure S10], with a median size of 19.3 kb ± 17.2 kb [see Additional file 12: Figure S11a] and a median variant count of 100 ± 0.5. Across these regions and depths of coverage, the median number of haplotypes observed was 3 [see Additional file 12: Figure S11b]. If genotype imputation and phasing were consistent across sequencing depths, then we would expect to observe either one (i.e. if all genotypes are homozygous) or two haplotypes (if one or more genotypes are heterozygous) for an individual in any given region. A median number of three haplotypes occurs when there is a single variant in the region that returns a different genotype or phase (i.e. 1|0 rather than 0|1) in one or more sequencing depths when compared to all other sequencing depths. The 5% upper tail of possible haplotypes is 8, for which we observed a median region size of 17 kb ± 22.5 kb, indicating that haplotypes with apparently systemic genotype or phasing inconsistencies across different sequencing depths were not a result of having derived from larger region sizes. There was no discernible difference in the distribution of median Z-scores relative to the median number of haplotypes identified for each region at any of the sequencing depths [see Additional file 12: Figure S11c]. This indicates that for regions that host potentially problematic haplotypes, there is no clearly observable consequence—i.e. there is no shrinkage of XPEHH values towards zero, or a shift towards extreme values.

Next, we investigated whether there was a significant difference in the mean sequencing depth within each region relative to the mean across the chromosome for each sample and depth of coverage. We binned the results by the median number of haplotypes observed across all samples within each region and regressed the Wilcox test p values from comparing mean region depth to mean chromosome depth, against the median haplotype count for each region (rounded up). The result indicated a highly significant (p < 0.001) difference in sequencing depth relative to the chromosome average for regions with eight or more haplotypes [see Additional file 1: Table S8 and Additional file 12: Figure S11d]. These results suggest that significant XPEHH results in genomic regions that have significantly reduced depth of coverage relative to the chromosome mean should be viewed with caution.

**Fig. 9:**
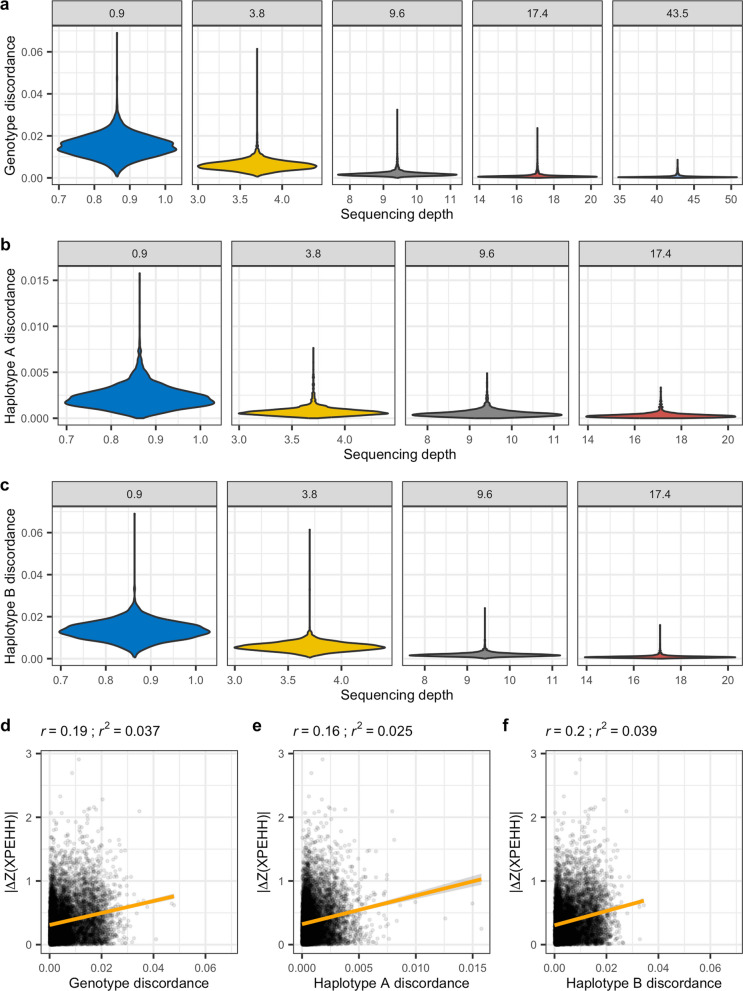
Imputation discordance relative to sequencing depth and XPEHH score divergence. **a**–**c** Mean sequencing depth and discordance were each calculated in 1-Mb windows along all the autosomes. **a** Genotype discordance was calculated for post-imputation genotypes at each sequencing depth relative to pre-imputation genotypes at 43.5X depth. **b**–**c** Haplotype discordance was calculated for each sequencing depth relative to each of the 43.5X haplotypes. The absolute difference in Z(XPEHH) values between those calculated at 43.5X and those calculated at lower depth datasets was plotted against **d** genotype discordance, **e** haplotype A discordance, and **f** haplotype B discordance

To further explore the relationship of sequencing depth with imputation and phasing errors, we calculated a number of metrics in 1-Mb windows along each of the autosomes and across samples, for each sequencing depth. These metrics included mean sequencing depth, genotype discordance between the 43.5X dataset pre-imputation (acting here as the ‘truth’ genotypes) and each other sequencing depth post-imputation, haplotype discordance relative to the 43.5X dataset post-imputation, and the median XPEHH Z-score. Values in the extreme tails of the local depth distribution (< 0.01 and > 0.99) were discarded to reduce any influence of stochastic extremes in sequencing coverage. Substantial differences in genotype discordance were observed at depths ≤ 3.8X compared to depths at ≥ 9.6X (Fig. 9a). A similar pattern was observed when comparing discordance in haplotypes A (Fig. 9b) and B (Fig. 9c) relative to the 43.5X haplotypes. To determine whether there was a relationship between genotype or haplotype discordance and XPEHH results, we plotted the absolute difference in XPEHH Z-scores between each sequencing depth relative to the 43.5X Z-scores against each discordance measure (Fig. 9d–f). The resulting correlation values revealed weak correlations (0.16 ≤ Pearson’s *r* ≤ 0.2), with *r*^*2*^ values from the linear regression indicating that discordance accounted for between 2.5 and 3.9% of the variance in Z-scores.

Pearson correlations between the 43.5X median XPEHH values in 1-Mb windows and those from each other sequencing depth were 0.66, 0.87, 0.89, and 0.91 for 0.9X, 3.8X, 9.6X, and 17.4X, respectively. Regressing these XPEHH values on depth of coverage calculated for the same windows, while accounting for the mean autosomal depth of coverage for each dataset, indicated a significant relationship that weakened with increasing sequencing depth, with t-statistic p values of 0.79, 4.37 × 10^–06^, 0.001, 0.079, and 0.33 for 0.9X, 3.8X, 9.6X, and 17.4X, respectively. The non-significant value at 0.9X is likely due to its lower variance in depth of coverage (*σ*^2^ = 0.001) compared to the other datasets (3.8X *σ*^2^ = 0.02, 9.6X *σ*^2^ = 0.13, 17.4X *σ*^2^ = 0.42, and 43.5X *σ*^2^ = 2.65). These results indicate that there is a correlation between sequencing depth and imputation accuracy. As we observed little difference in imputation accuracy across the three reference panels used, we believe that the correlation with sequencing depth is likely causal. However, in the absence of more extensive data to further explore the impact of reference panel composition on imputation accuracy, we cannot rule out reference panel composition as the major causal factor.

## Discussion

First, we have demonstrated that saliva-derived DNA is suitable for WGS and subsequent analysis of SNVs. To date, most canine studies of the genome involve DNA extracted from blood, and this is the preferred sample type outlined in the Dog10K project [28]. Previous studies have demonstrated that both saliva and buccal-derived DNA are comparable in terms of efficacy to blood-derived DNA for array-based genome-wide association studies [29, 30]. Saliva collection is less invasive than blood sampling and can be performed by dog owners, highlighting the feasibility of client-based sample collection outside of the clinical setting.

Our results indicate that genotype imputation of low-pass sequencing data can be performed with a high degree of accuracy, although two species-agnostic limitations require attention. The first of these concerns rare variants, specifically those with a MAF ≤ 0.01, as previously reported and illustrated here. When a variant is observed at low frequency in the reference panel, it becomes statistically challenging to establish the associated haplotype background [10]. Variant allele frequencies can be manipulated through careful curation of the reference panel and there will be a trade-off in this regard with respect to the size of the panel. For instance, panels that comprise thousands of individuals could result in an increase in rare variants due to increased genetic diversity, but also through disproportionate breed representation in the panel’s composition. Our imputation results are broadly consistent regardless of whether the full reference panel or a subset was used, and it is noteworthy that these panels share similar allele frequency distributions in spite of a near four-fold difference in size between the full panel and its two subsets. It is also worth noting at this point that the GLIMPSE IMPUTE INFO score has been reported to be positively correlated with variant MAF [7], and thus care should be taken if this is used for filtering, as it may result in removal of low-frequency variants.

The second limitation relates to the accuracy of phasing. Our results indicate that, although genotype imputation enabled cross-validation of SNVs associated with the chocolate coat phenotype, using an approach that tests each SNV independently (GEMMA), the haplotype-based XP-EHH method was considerably less sensitive. This is surprising given the extensive linkage disequilibrium observed among pedigree dogs, which results in large haplotype blocks, limited haplotype block diversity due to selective breeding, and the ability to use a haplotype-based method for imputation. This loss of sensitivity could be due to the stochastic nature of imputation, or a consequence of haplotype degradation arising from phasing errors during imputation, which typically occurs as a result of constraints in the available haplotypes in the reference panel. As such, a large and diverse reference panel is an essential resource to facilitate the uptake of low-pass sequencing while maintaining confidence in imputation accuracy. In this regard, the increasing adoption of long-read sequencing [31] and use of haplotagging methods [32] when developing a reference panel will significantly improve phasing accuracy and downstream imputation. Our analyses indicate that erroneous haplotypes may, to some extent, result from localised reductions in depth of coverage relative to the average coverage across the chromosome. Further investigation is warranted to investigate this in more detail, such that it can be mitigated in future studies.

The high genotype concordance that we observed for non-rare variants implies that a stratified approach to imputing missing genotypes in a reference panel may be appropriate and is worth investigating. Sequentially imputing variants stratified by MAF, ranked by increasing rarity, could improve the imputation accuracy of rare variants. Similar multi-step approaches have previously been demonstrated in humans [33], cattle [34], and sheep [35].

A substantial body of work is available on low-pass sequencing and imputation, predominantly assessing its application in livestock breeding programmes, where it is not uncommon to process data for tens of thousands of animals and to leverage detailed pedigree information to improve imputation concordance [36, 37]. In such cases, the application of low-pass sequencing is generally undertaken with the intent to derive genomic estimated breeding values to support genetic selection for traits of interest. Unlike in production animals, companion animal genomics rarely has the opportunity to leverage pedigree information to help guide imputation.

## Conclusions

Imputation accuracy is contingent on a comprehensive reference panel of haplotypes that are representative of population diversity. Our results indicate negligible differences in imputation performance between reference panels comprising small or large numbers of haplotypes when the variant allele frequencies of the panels are broadly consistent. This indicates that allelic diversity is more important than the number of haplotypes in the reference panel. Due consideration needs to be given on intended downstream analyses before generating low-pass sequence data. We found that sequencing to 3.6X depth captured 95% of the genome with a mean genotype imputation concordance of 95%. At this depth, the results of a single-marker association analysis were highly correlated (*r* ≥ 0.97) with those from the same library sequenced to 43.5X depth. XP-EHH results were less well correlated between the 43.5X dataset and those sequenced at lower depths, and further investigation of a putative false positive highlighted genotype and phasing inconsistencies across the depths. As such, for studies focused on single-marker based analyses, we recommend sequencing to at least 3.6X. In contrast, given the high correlation (*r* = 0.91) in median XPEHH values between 17.4X and 43.5X datasets and the absence of a significant correlation between local depth of coverage and XPEHH values at 17.4X, we conclude that robust haplotype-based analyses require at least 17.4X depth of coverage. These considerations are especially relevant for studies where multi-generational data (e.g. genotype and pedigree) are unavailable, as these would otherwise reduce imputation inconsistencies that arise due to poorly resolved haplotypes.

### Supplementary Information

**Additional file 1: Table S1. **Sequencing coverage summary statistics. Sample ID, gender, coat colour, and sequencing summary statistics for each library sequenced. **Table S2. **Summary of variant records in VCF files following each filtering step described in Additional file 2: Figure S1. Breakdown of variants tagged with different filtering criteria, and subsequently remaining after each filtering step. **Table S3.** Reference panels for phasing and imputation. List of dog ID and breeds comprising the full reference panel, with those included in panels 1 and 2 indicated, respectively. **Table S4.** Summary of variant counts binned by allele frequency, those reported with different depths are derived from the full panel. Breakdown of variant counts per allele frequency bin for the full reference panel, and subset panels 1 and 2. Also provided are variant counts in the imputed data at each sequencing depth based on using the full reference panel for phasing and imputation. **Table S5.** Linear model coefficient estimates after fitting imputed genotype *r*^2^ to MAF, with depth and panel as covariates: lm(*r*^2^ ~ MAF + depth + panel). Summary table of coefficient estimates after fitting a linear model of imputed genotype *r*^2^ to MAF, including depth and reference panel as covariates. **Table S6.** Linear model coefficient estimates after fitting SNV imputed dosage *r*^2^ to depth, with panel as a covariate: lm(*r*^2^ ~ depth + panel). Summary table of coefficient estimates after fitting a linear model of SNV imputed dosage *r*^2^ to depth, including reference panel as a covariate. **Table S7.** Summary of mismatch rates for SNVs arising from different reference panels at each depth of coverage tested. Table of mismatch rates for homozygous reference (RR), heterozygous reference (RA), and homozygous alternate (AA) genotypes of SNVs when comparing each depth of coverage to the genotypes from the 43.5X depth dataset, derived from imputation with each of the reference panels. **Table S8.** Linear model coefficient estimates after fitting paired Wilcox test p values from comparing region and chromosome depth to region median haplotype count: lm(p ~ haps). Summary table of coefficient estimates after fitting linear model of paired Wilcox test p values from comparing region and chromosome depth to region median haplotype count.

**Additional file 2: Figure S1.** Workflow to generate the reference panel for use in imputation. Strelka genome variant call files (g.VCFs) for 1706 dogs were jointly genotyped, identifying more than 1.4 billion variants. After applying a series of filters, a final dataset of 1021 samples and 9.2M variant records was retained [see Additional file 1: Table S1]. The genotypes were recorded for each chromosome in separate variant call files (VCF).

**Additional file 3: Figure S2. **Workflow illustrating processing of raw sequence data through to imputation. DNA extracted from saliva of 30 Labrador retrievers was sequenced to 0.9X, 3.8X, and 43.5X depths of coverage, using the same library preparations for each sequencing run. The aligned 43.5X data was also down-sampled *in silico* to 9.6X and 17.4X depths of coverage. The GLIMPSE workflow was applied to impute and phase genotypes at variants in the full reference panel [see Additional file 2: Figure S1], in addition to two subsets of that reference panel which primarily differed by the number of Labrador retrievers included. Briefly, this involves calling genotypes from alignment files (BAM) using mpileup to generate pre-imputation variant call files (VCF). These are then phased and imputed with GLIMPSE, using a reference panel. Genotypes with low probabilities (GP < 0.95) are masked, and phased genotypes recorded in post-imputation VCF.

**Additional file 4: Figure S3.** Workflow illustrating data inputs for concordance and association analyses. **a** Concordance analyses were performed on pre-imputation VCF genotypes from each sequencing depth relative to those at 43.5X depth, using bcftools, and on post-imputation VCF genotypes using GLIMPSE. Analysis of post-imputation genotypes leverages the allele frequencies of the reference panel used for imputation to bin the data. **b** Association analyses were performed on post-imputation genotypes, applying a single-marker approach, GEMMA, and a haplotype-based approach, XPEHH.

**Additional file 5: Figure S4.** Manhattan plot of GEMMA results following imputation with the full reference panel. Chromosomes are plotted in alternating colours (orange, blue), with the chromosome number indicated at the top of the figure. The location of the *TYRP1* locus on chromosome 11 is indicated with a vertical line.

**Additional file 6: Figure S5.** Manhattan plot of GEMMA results following imputation with the subset panel 1. Chromosomes are plotted in alternating colours (orange, blue), with the chromosome number indicated at the top of the figure. The location of the *TYRP1* locus on chromosome 11 is indicated with a vertical line.

**Additional file 7: Figure S6.** Manhattan plot of GEMMA results following imputation with the subset panel 2. Chromosomes are plotted in alternating colours (orange, blue), with the chromosome number indicated at the top of the figure. The location of the *TYRP1* locus on chromosome 11 is indicated with a vertical line.

**Additional file 8: Figure S7.** Manhattan plot of XPEHH Z-scores following imputation with the full reference panel. Chromosomes are plotted in alternating colours (orange, blue), with the chromosome number indicated at the top of the figure. The location of the *TYRP1* locus on chromosome 11 is indicated with a vertical line.

**Additional file 9: Figure S8.** Manhattan plot of XPEHH Z-scores following imputation with the subset panel 1. Chromosomes are plotted in alternating colours (orange, blue), with the chromosome number indicated at the top of the figure. The location of the *TYRP1* locus on chromosome 11 is indicated with a vertical line.

**Additional file 10: Figure S9.** Manhattan plot of XPEHH Z-scores following imputation with the subset panel 2. Chromosomes are plotted in alternating colours (orange, blue), with the chromosome number indicated at the top of the figure. The location of the *TYRP1* locus on chromosome 11 is indicated with a vertical line.

**Additional file 11: Figure S10.** Chromosome histograms showing distribution of genomic regions with excessive haplotype counts. Histograms indicate the count of regions with ≥ 8 haplotypes across sequencing depths for a given dog and based on randomly sampling 10K regions across autosomes.

**Additional file 12: Figure S11. **Analysis of haplotype counts with respect to XPEHH values and deviations in depth. **a** Histogram of genomic region size after randomly sampling 10,000 genomic regions. **b** Histogram of the number of possible haplotypes identified per dog across the different sequencing depths for these 10,000 regions. **c** Box plots illustrating the mean XPEHH Z-score distribution, relative to the number of possible haplotypes for these 10,000 regions. **d** Box plots illustrating the median paired Wilcox test p values relative to median haplotype number across the 10,000 regions.

## Data Availability

Raw sequencing reads for the dogs sequenced in this study are available from the European Nucleotide Archive (https://www.ebi.ac.uk/ena), under project accession code PRJEB59274. The reference panel was generated from publicly available sequence data and will be described in detail elsewhere (Wengang Zhang et al. manuscript in preparation). Code underpinning the pipeline (BAGPIPE) used for aligning sequence data and calling variants BAGPIPE is available at https://bitbucket.org/renzo_tale/bagpipe. Code used to process the data is available at https://bitbucket.org/gibberwocky/cautionary_tale. The data supporting this study is available at https://doi.org/10.7488/9b751f11-266c-4c5d-9e57-d84d8c102324.
