# Supplementary figures and images for: A cautionary tale of low-pass sequencing and imputation with respect to haplotype accuracy
Source: Genet Sel Evol. 2024 Jan 12;56:6. doi: 10.1186/s12711-024-00875-w (PMC10785484; doi:10.1186/s12711-024-00875-w)

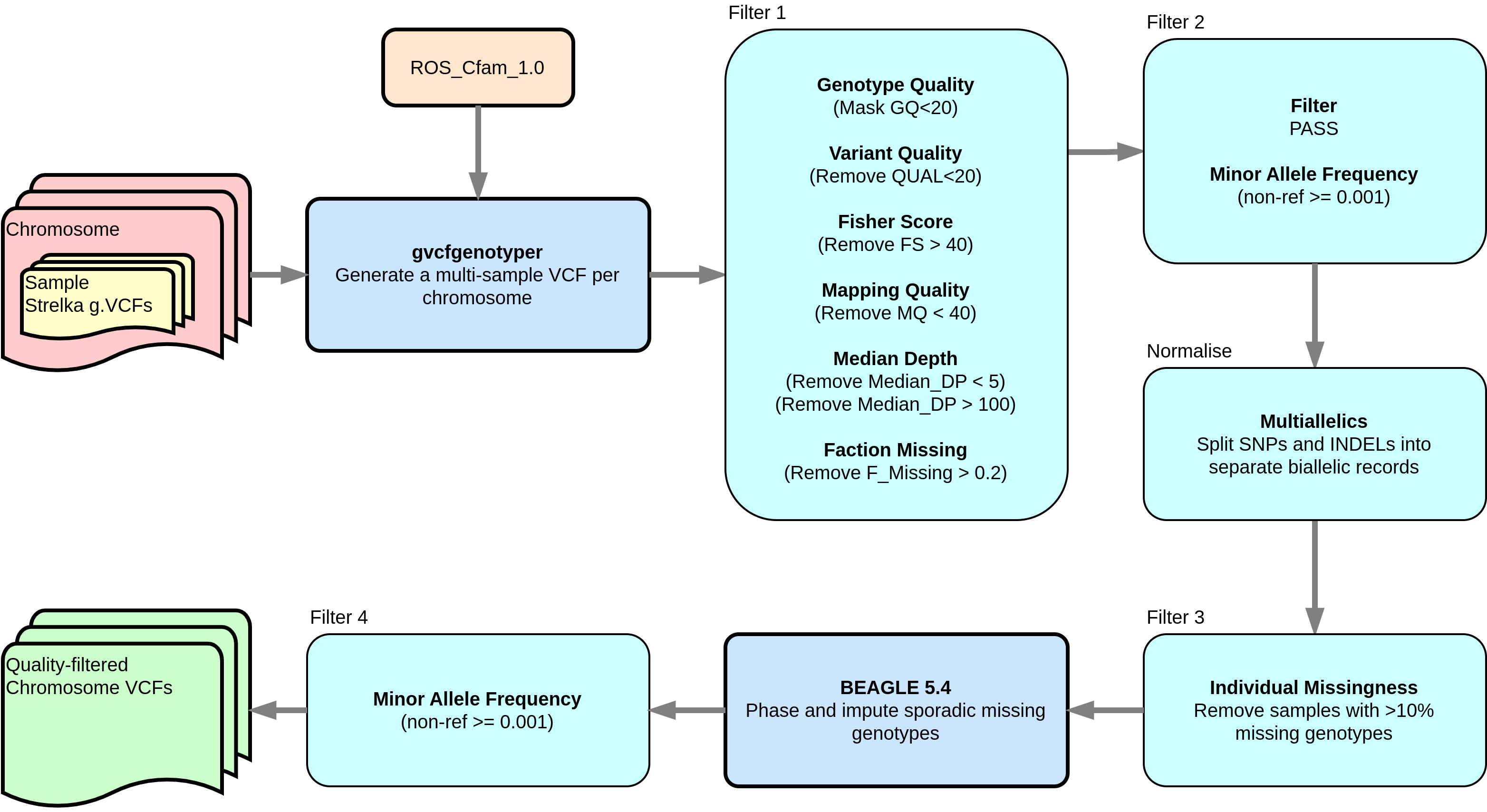

Supplement: Supplementary file 2 — Additional file 2: Figure S1. Workflow to generate the reference panel for use in imputation. Strelka genome variant call files (g.VCFs) for 1706 dogs were jointly genotyped, identifying more than 1.4 billion variants. After applying a series of filters, a final dataset of 1021 samples and 9.2M variant records was retained [see Additional file 1: Table S1]. The genotypes were recorded for each chromosome in separate variant call files (VCF). [file 12711_2024_875_MOESM2_ESM.png]

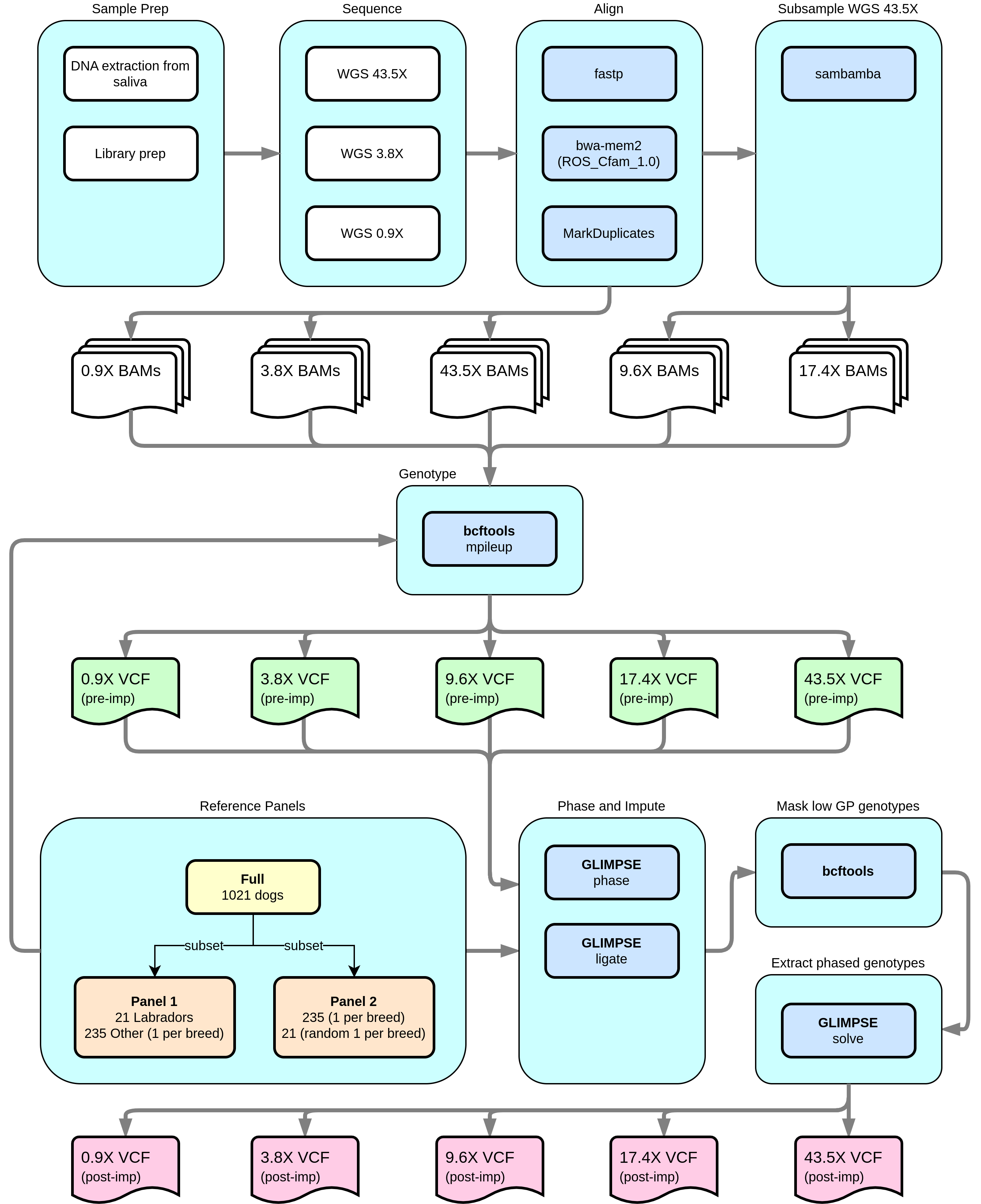

Supplement: Supplementary file 3 — Additional file 3: Figure S2. Workflow illustrating processing of raw sequence data through to imputation. DNA extracted from saliva of 30 Labrador retrievers was sequenced to 0.9X, 3.8X, and 43.5X depths of coverage, using the same library preparations for each sequencing run. The aligned 43.5X data was also down-sampled in silico to 9.6X and 17.4X depths of coverage. The GLIMPSE workflow was applied to impute and phase genotypes at variants in the full reference panel [see Additional file 2: Figure S1], in addition to two subsets of that reference panel which primarily differed by the number of Labrador retrievers included. Briefly, this involves calling genotypes from alignment files (BAM) using mpileup to generate pre-imputation variant call files (VCF). These are then phased and imputed with GLIMPSE, using a reference panel. Genotypes with low probabilities (GP < 0.95) are masked, and phased genotypes recorded in post-imputation VCF. [file 12711_2024_875_MOESM3_ESM.png]

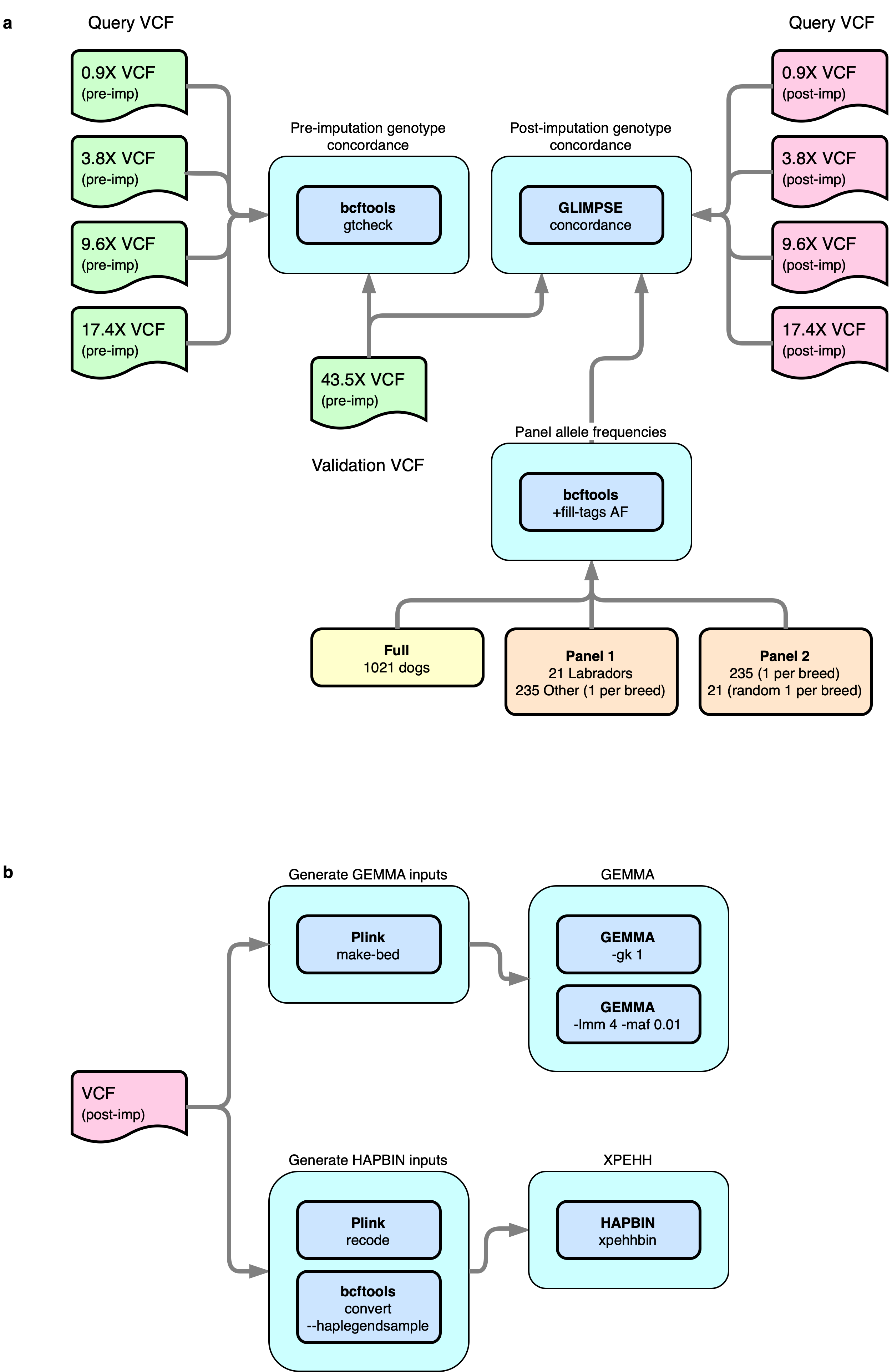

Supplement: Supplementary file 4 — Additional file 4: Figure S3. Workflow illustrating data inputs for concordance and association analyses. a Concordance analyses were performed on pre-imputation VCF genotypes from each sequencing depth relative to those at 43.5X depth, using bcftools, and on post-imputation VCF genotypes using GLIMPSE. Analysis of post-imputation genotypes leverages the allele frequencies of the reference panel used for imputation to bin the data. b Association analyses were performed on post-imputation genotypes, applying a single-marker approach, GEMMA, and a haplotype-based approach, XPEHH. [file 12711_2024_875_MOESM4_ESM.png]

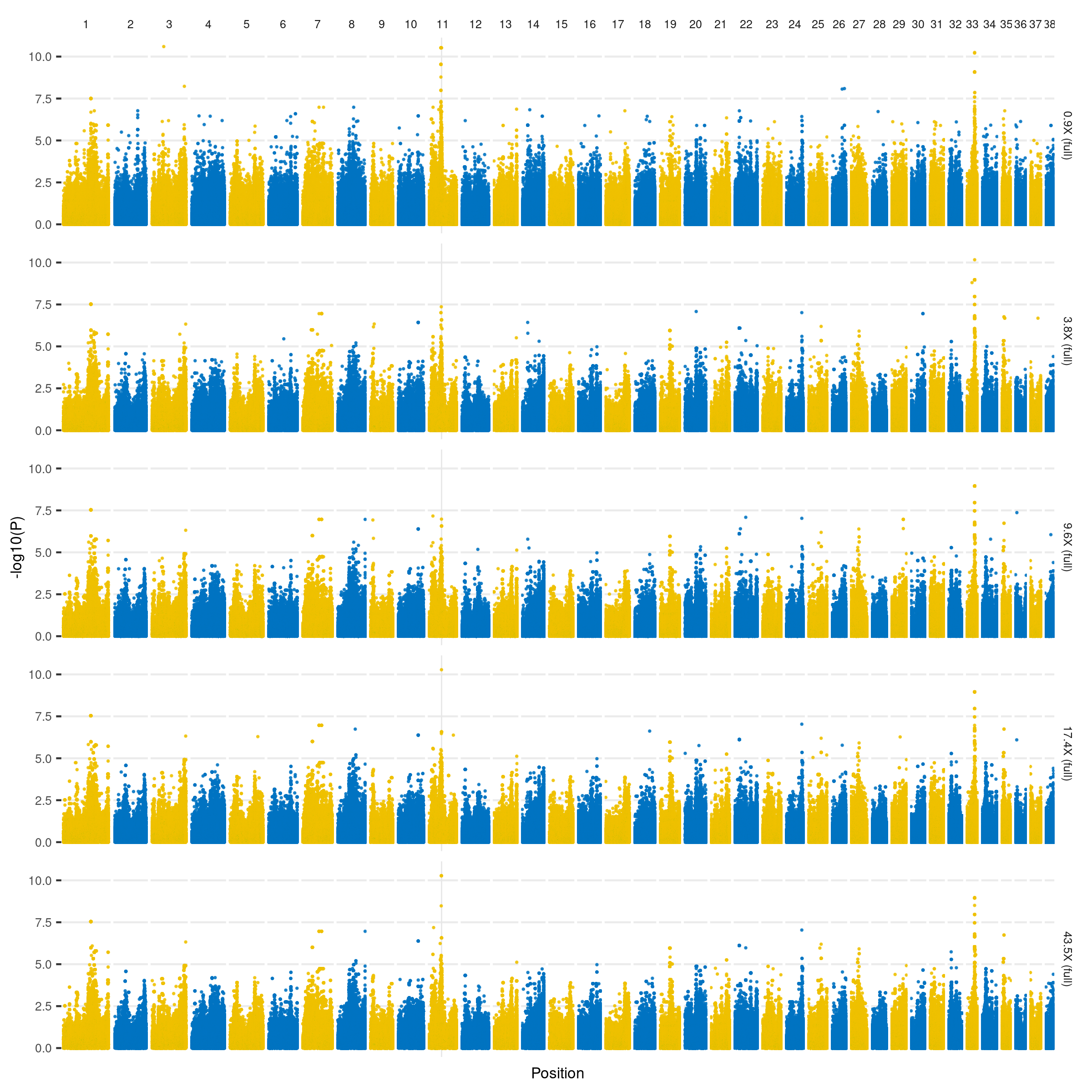

Supplement: Supplementary file 5 — Additional file 5: Figure S4. Manhattan plot of GEMMA results following imputation with the full reference panel. Chromosomes are plotted in alternating colours (orange, blue), with the chromosome number indicated at the top of the figure. The location of the TYRP1 locus on chromosome 11 is indicated with a vertical line. [file 12711_2024_875_MOESM5_ESM.png]

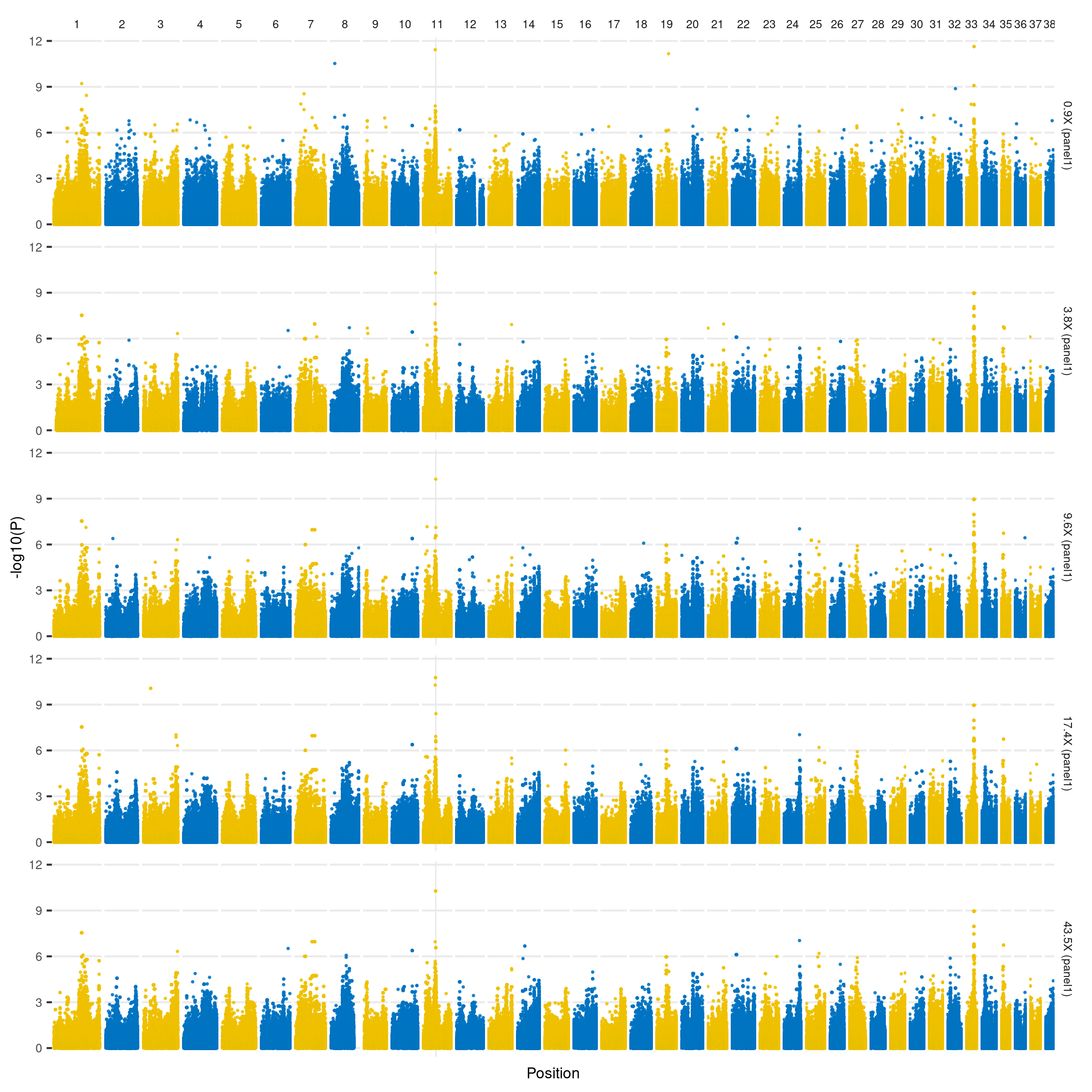

Supplement: Supplementary file 6 — Additional file 6: Figure S5. Manhattan plot of GEMMA results following imputation with the subset panel 1. Chromosomes are plotted in alternating colours (orange, blue), with the chromosome number indicated at the top of the figure. The location of the TYRP1 locus on chromosome 11 is indicated with a vertical line. [file 12711_2024_875_MOESM6_ESM.png]

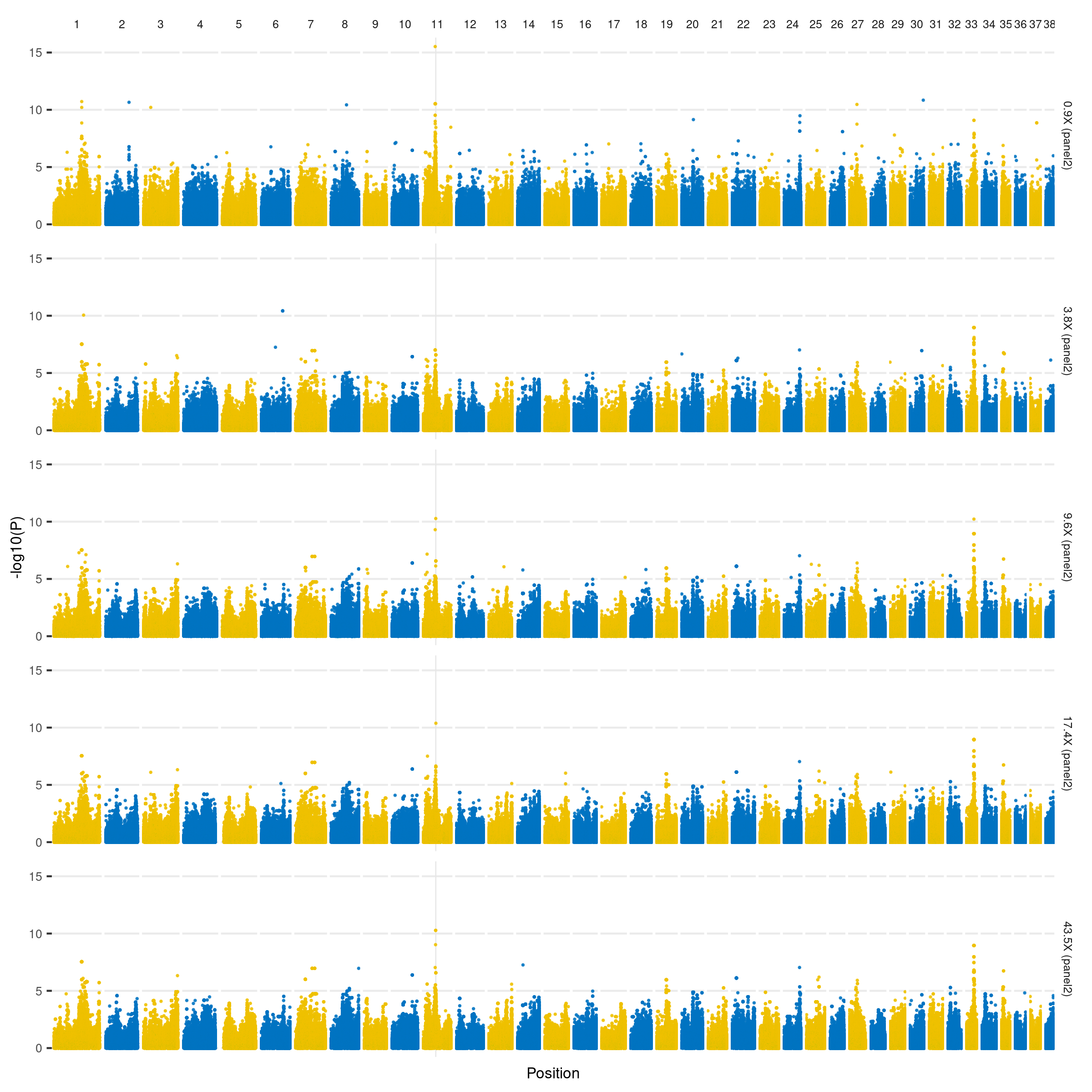

Supplement: Supplementary file 7 — Additional file 7: Figure S6. Manhattan plot of GEMMA results following imputation with the subset panel 2. Chromosomes are plotted in alternating colours (orange, blue), with the chromosome number indicated at the top of the figure. The location of the TYRP1 locus on chromosome 11 is indicated with a vertical line. [file 12711_2024_875_MOESM7_ESM.png]

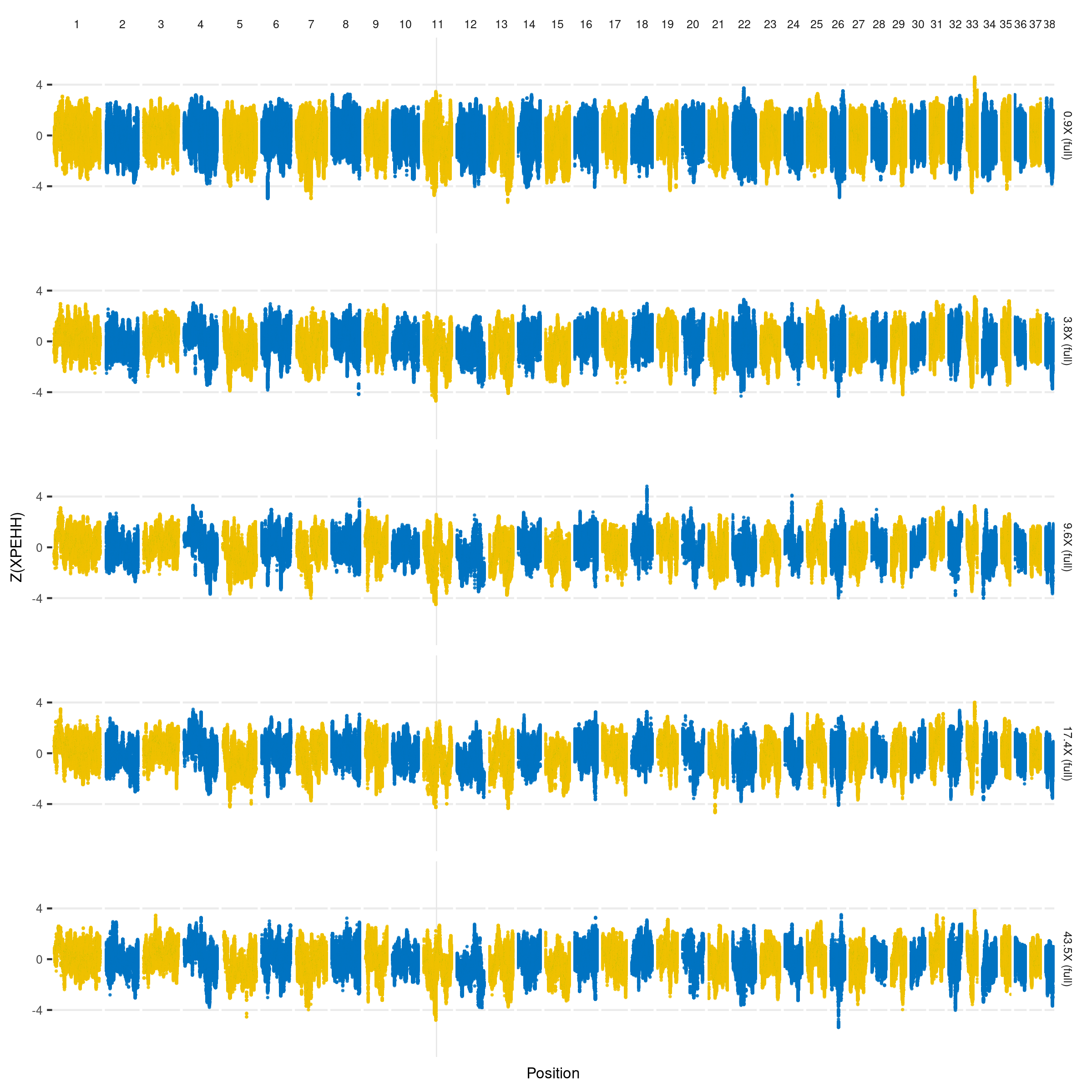

Supplement: Supplementary file 8 — Additional file 8: Figure S7. Manhattan plot of XPEHH Z-scores following imputation with the full reference panel. Chromosomes are plotted in alternating colours (orange, blue), with the chromosome number indicated at the top of the figure. The location of the TYRP1 locus on chromosome 11 is indicated with a vertical line. [file 12711_2024_875_MOESM8_ESM.png]

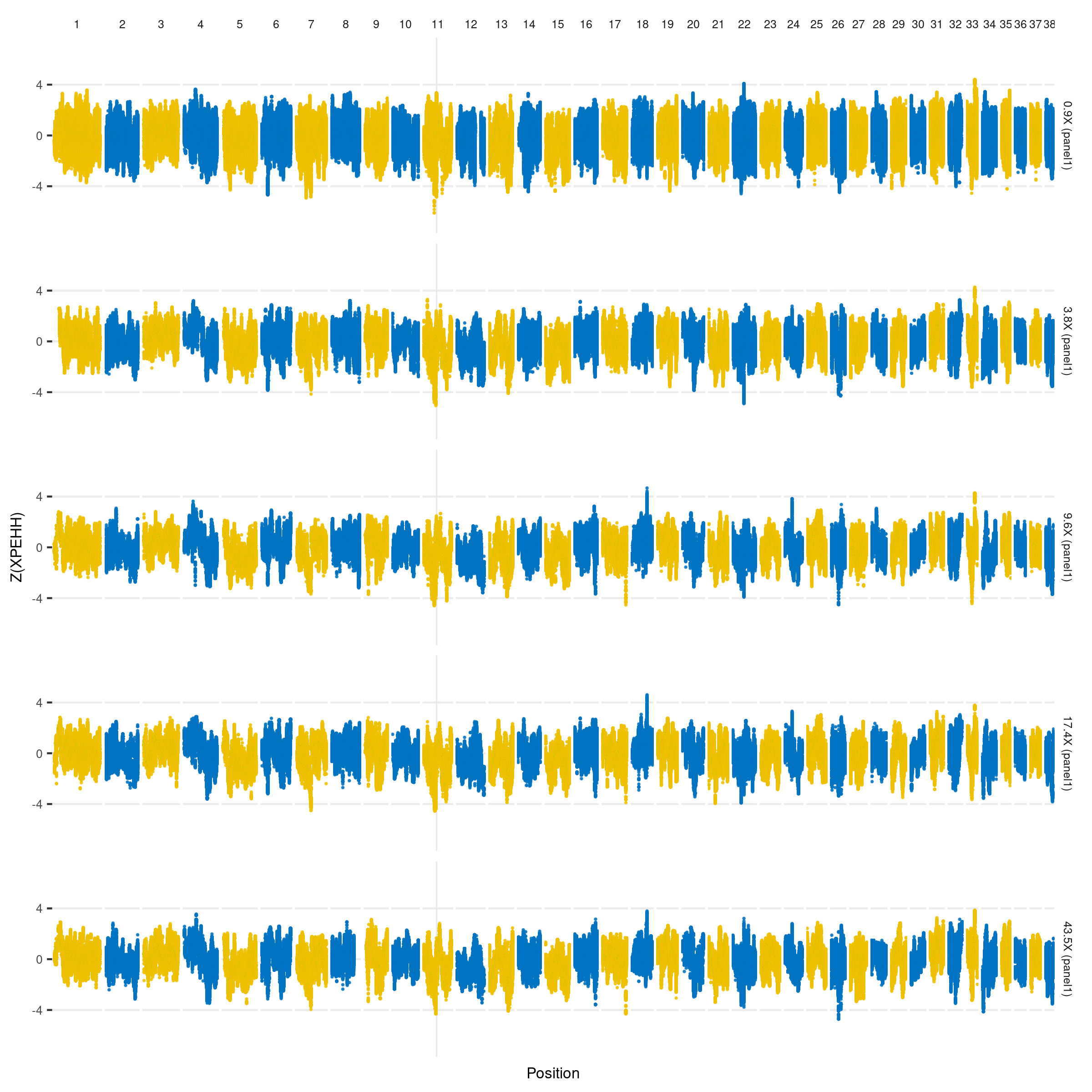

Supplement: Supplementary file 9 — Additional file 9: Figure S8. Manhattan plot of XPEHH Z-scores following imputation with the subset panel 1. Chromosomes are plotted in alternating colours (orange, blue), with the chromosome number indicated at the top of the figure. The location of the TYRP1 locus on chromosome 11 is indicated with a vertical line. [file 12711_2024_875_MOESM9_ESM.png]

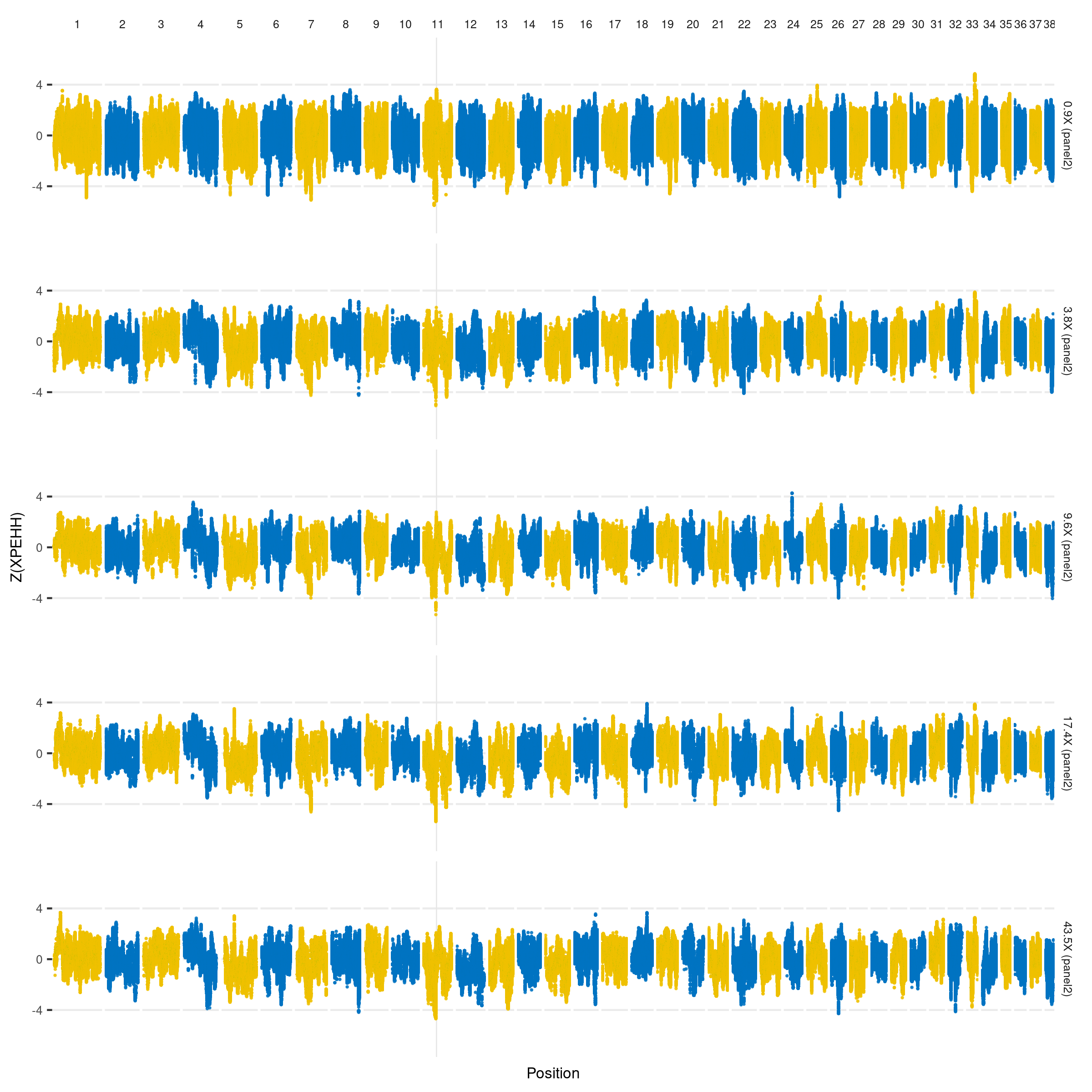

Supplement: Supplementary file 10 — Additional file 10: Figure S9. Manhattan plot of XPEHH Z-scores following imputation with the subset panel 2. Chromosomes are plotted in alternating colours (orange, blue), with the chromosome number indicated at the top of the figure. The location of the TYRP1 locus on chromosome 11 is indicated with a vertical line. [file 12711_2024_875_MOESM10_ESM.png]

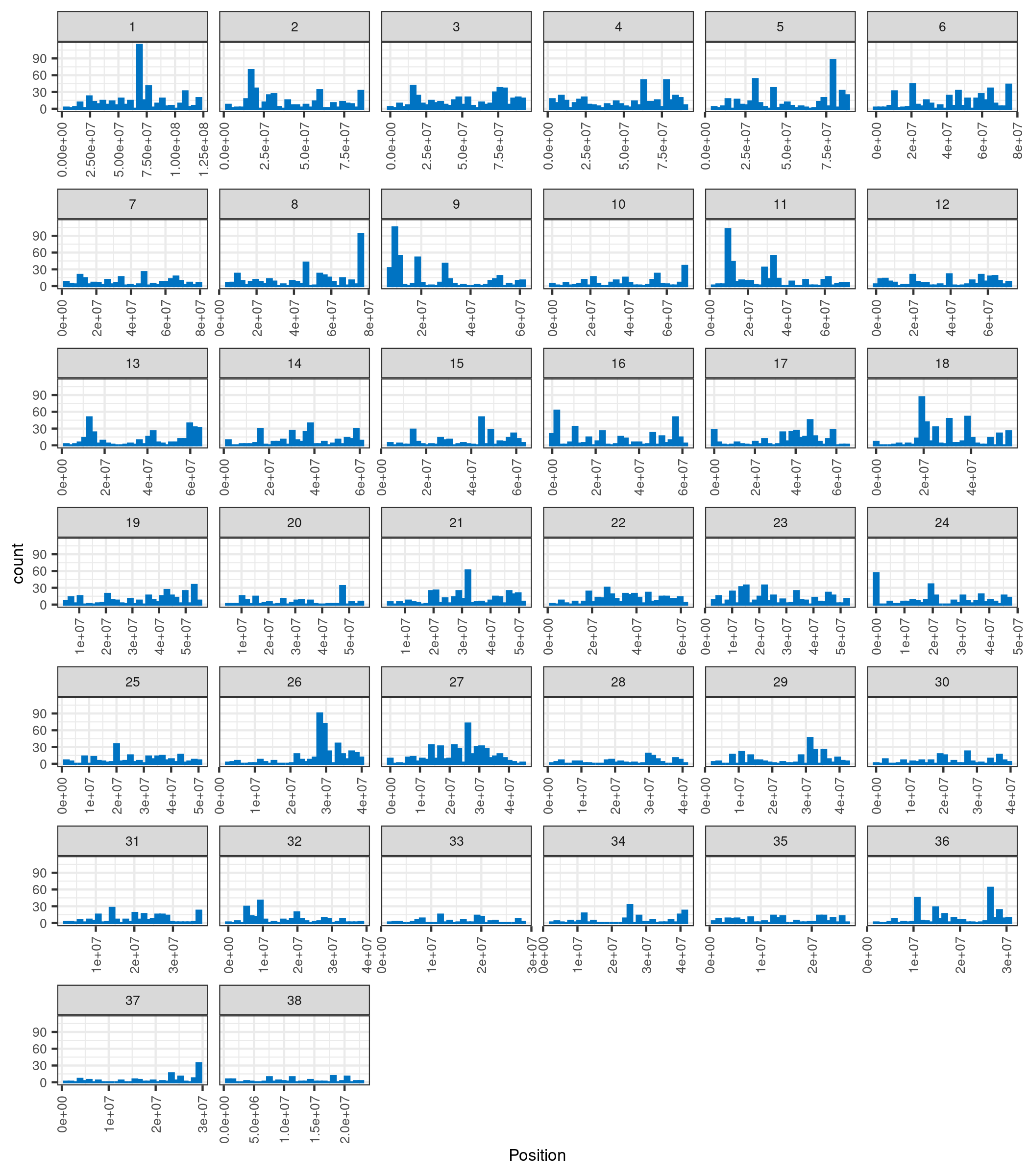

Supplement: Supplementary file 11 — Additional file 11: Figure S10. Chromosome histograms showing distribution of genomic regions with excessive haplotype counts. Histograms indicate the count of regions with ≥ 8 haplotypes across sequencing depths for a given dog and based on randomly sampling 10K regions across autosomes. [file 12711_2024_875_MOESM11_ESM.png]

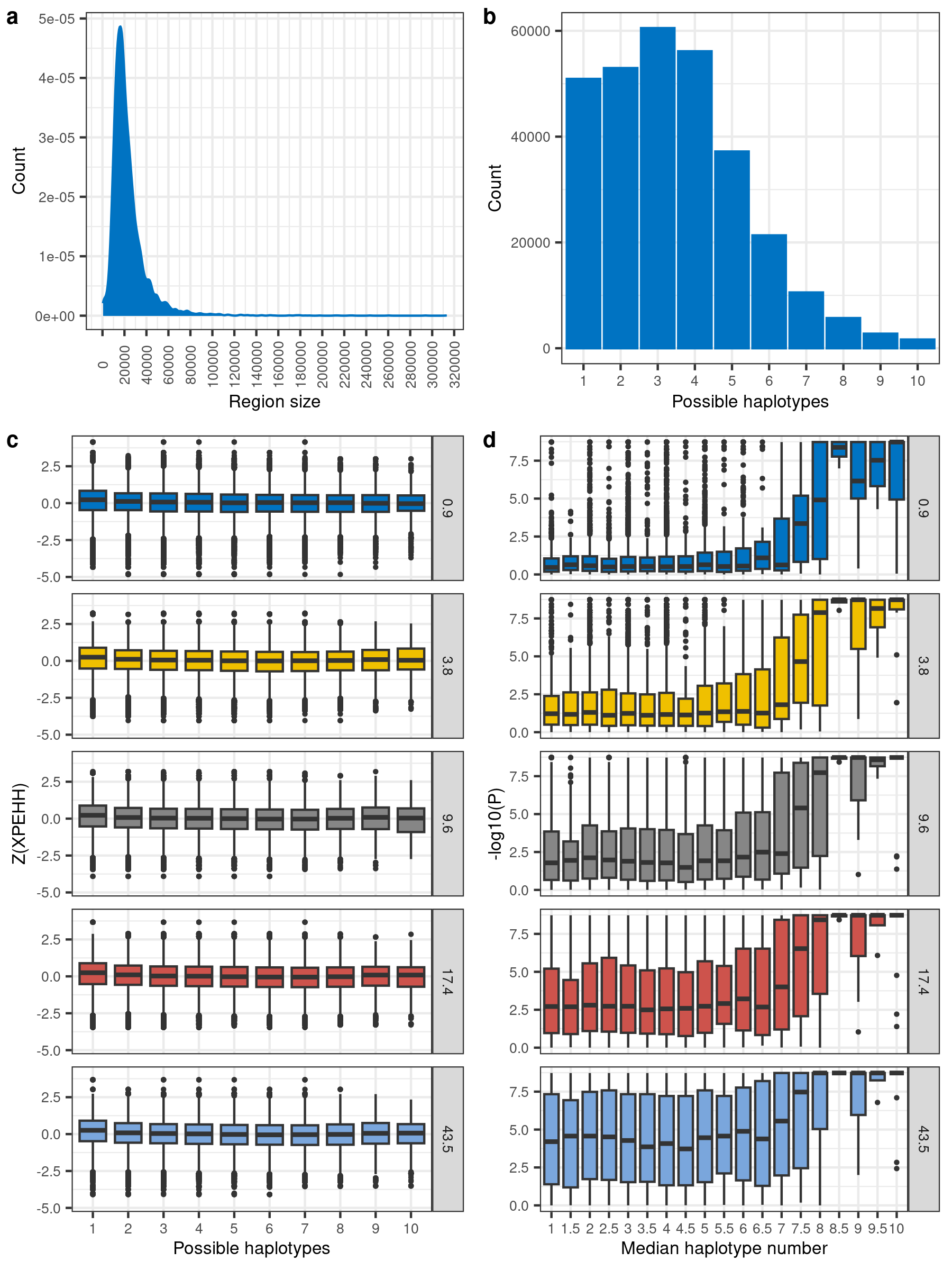

Supplement: Supplementary file 12 — Additional file 12: Figure S11. Analysis of haplotype counts with respect to XPEHH values and deviations in depth. a Histogram of genomic region size after randomly sampling 10,000 genomic regions. b Histogram of the number of possible haplotypes identified per dog across the different sequencing depths for these 10,000 regions. c Box plots illustrating the mean XPEHH Z-score distribution, relative to the number of possible haplotypes for these 10,000 regions. d Box plots illustrating the median paired Wilcox test p values relative to median haplotype number across the 10,000 regions. [file 12711_2024_875_MOESM12_ESM.png]
